# Spacer Layer Thickness Dependence of the Giant Magnetoresistance in Electrodeposited Ni-Co/Cu Multilayers

**DOI:** 10.3390/nano12234276

**Published:** 2022-12-01

**Authors:** Sándor Zsurzsa, Moustafa El-Tahawy, László Péter, László Ferenc Kiss, Jenő Gubicza, György Molnár, Imre Bakonyi

**Affiliations:** 1 Institute for Solid State Physics and Optics, Wigner Research Centre for Physics, Konkoly-Thege út 29-33, H-1121 Budapest, Hungary; 2 Department of Materials Physics, Eötvös Loránd University, Pázmány Péter Sétány 1/A, H-1117 Budapest, Hungary; 3 Physics Department, Faculty of Science, Tanta University, Tanta 31527, Egypt; 4 Institute for Technical Physics and Materials Science, Energy Research Centre, Konkoly-Thege út 29-33, H-1121 Budapest, Hungary

**Keywords:** electrodeposition, multilayers, X-ray diffraction study, magnetic properties, giant magnetoresistance

## Abstract

Electrodeposited Ni_65_Co_35_/Cu multilayers were prepared with Cu spacer layer thicknesses between 0.5 nm and 7 nm. Their structure and magnetic and magnetoresistance properties were investigated. An important feature was that the Cu layers were deposited at the electrochemically optimized Cu deposition potential, ensuring a reliable control of the spacer layer thickness to reveal the true evolution of the giant magnetoresistance (GMR). X-ray diffraction indicated satellite reflections, demonstrating the highly coherent growth of these multilayer stacks. All of the multilayers exhibited a GMR effect, the magnitude of which did not show an oscillatory behavior with spacer layer thickness, just a steep rise of GMR around 1.5 nm and then, after 3 nm, it remained nearly constant, with a value around 4%. The high relative remanence of the magnetization hinted at the lack of an antiferromagnetic coupling between the magnetic layers, explaining the absence of oscillatory GMR. The occurrence of GMR can be attributed to the fact that, for spacer layer thicknesses above about 1.5 nm, the adjacent magnetic layers become uncoupled and their magnetization orientation is random, giving rise to a GMR effect. The coercive field and magnetoresistance peak field data also corroborate this picture: with increasing spacer layer thickness, both parameters progressively approached values characteristic of individual magnetic layers. At the end, a critical analysis of previously reported GMR data on electrodeposited Ni-Co/Cu multilayers is provided in view of the present results. A discussion of the layer formation processes in electrodeposited multilayers is also included, together with a comparison with physically deposited multilayers.

## 1. Introduction

### 1.1. Giant Magnetoresistance in Multilayers

The giant magnetoresistance (GMR) effect [1,2] was discovered in nanoscale metallic magnetic/non-magnetic multilayer films in 1988. GMR multilayers with several element combinations, such as Fe/Cr or Co/Cu, could be prepared by various physical deposition methods, such as molecular-beam epitaxy, evaporation, and sputtering. It was discovered that, at certain thicknesses of the non-magnetic (so-called spacer) layer [3,4], the resistivity of the multilayer stack is much larger in a zero magnetic field than in high magnetic fields. The field-induced resistivity change of such multilayers was found to be as high as 50% at room temperature, as opposed to the resistivity change, of at most 5%, known in bulk ferromagnets due to the anisotropic magnetoresistance (AMR) effect [5]. The large zero-field resistivity was a consequence of the antiparallel alignment of the adjacent layer magnetizations due to an antiferromagnetic (AF) exchange coupling between them [1,2]. Namely, under such conditions, the conduction electrons undergo a strong spin-dependent scattering when traveling through the non-magnetic spacer from one magnetic layer to a neighboring magnetic layer with an opposite magnetization orientation. On the other hand, in sufficiently large magnetic fields, the magnetizations of all magnetic layers are aligned parallel and the above-mentioned spin-dependent scattering events do not occur; therefore, the resistivity will be much smaller. The resistivity change between the antiparallel aligned state in zero magnetic field and the magnetically aligned state of the multilayer in a saturating magnetic field is the GMR effect.

Magnetoresistive (MR) sensors based on the AMR effect of ferromagnetic (FM) metals and alloys have long been in use [5,6] and a great prospect opened in the field of MR sensors by the availability of the much larger GMR effect. MR sensors are simple magnetic field detectors with a wide range of applications, such as rotation speed meters and current sensors, and they are also used extensively in microelectronics, the automotive industry and biology [6,7,8,9,10,11,12,13,14]. The major breakthrough of the GMR sensors was their application in the read head of hard disk drives [15], which has revolutionized the storage density well beyond the limits achieved on the basis of the previously used inductive read heads. In fact, the discovery of the GMR effect has paved the way to the advent of so-called spintronics [9,13], in which the electron spin is the key feature, rather than the electron charge in electronics.

Due to the permanent and widespread interest in GMR sensor applications, there is on-going research being conducted to improve the magnetoresistive properties of such multilayers by studying the influence of buffer layers [16], spacer layer materials [17], or surfactants [18] on layer growth. 

A key feature of the GMR effect in magnetic/non-magnetic multilayers is the oscillatory behavior [3,4]: the magnitude of the GMR effect oscillates as a function of the thickness of the non-magnetic spacer layer. This behavior has been well demonstrated for many multilayer systems produced by physical deposition methods with various magnetic and non-magnetic element or alloy combinations. The origin of the oscillatory GMR [3,4] is the alternation of FM and AF couplings between the adjacent magnetic layers with varying spacer thickness. At thicknesses corresponding to AF coupling, i.e., when the magnetizations of the adjacent magnetic layers are aligned antiparallel in zero magnetic field, the resistivity of the multilayer stack is large and, therefore, a maximum of the GMR effect occurs. On the other hand, at spacer thicknesses with FM coupling, i.e., with a parallel alignment of the magnetizations of all layers, the zero-field resistivity is smaller than for the antiparallel alignment and, therefore, a zero or at most a very small GMR effect occurs. The oscillation period is of the order of 1 nm and its precise value is determined by the matching of the electronic density of states of the constituent magnetic and non-magnetic metals [19,20,21].

It should be noted that the presence of an AF coupling between adjacent magnetic layers is not a prerequisite for the occurrence of a GMR effect. Namely, any misalignment of the adjacent layer magnetizations, irrespective of the presence or absence of any magnetic coupling between them, results in a GMR effect [22], the magnitude of which depends on the degree of misalignment. Evidently, the misalignment is the largest for the antiparallel alignment which occurs, e.g., for the case of an AF coupling. The existence of an orthogonal coupling was also considered for explaining the reduced GMR effect in some specially processed sputtered Co/Cu multilayers [23]. Theoretical modeling [22] revealed that the GMR effect reduces from the possible maximum value for the antiparallel alignment by about a factor of two for the case of the orthogonal coupling, just as observed in the experiment [23].

Along this line of thought, it can be concluded that one can also observe a GMR effect for magnetic/non-magnetic multilayers in which there is no coupling between the magnetizations of the adjacent magnetic layers. Namely, in the lack of any coupling, the layer magnetizations are randomly aligned in the layer planes, and even the layer magnetizations can be in a multidomain state [24]. Under such circumstances, it can frequently occur that in two neighboring magnetic layers, the layer magnetizations are not parallel aligned in a certain region in zero magnetic field and, then, this region immediately contributes a GMR effect once a magnetic field brings the multilayer into magnetic saturation.

### 1.2. Electrodeposited Multilayers with GMR

In addition to the above-mentioned physical deposition methods, it was demonstrated by Alper et al. [25,26] in 1993 that the cost-efficient and simple technique of electrodeposition can also be tailored to a level that enables the preparation of magnetic/non-magnetic multilayers exhibiting the GMR phenomenon. The study of GMR in electrodeposited multilayers has considerably expanded since that time as summarized in several reviews [27,28,29,30,31,32,33] and has remained an active area of research [34,35,36,37,38,39,40,41,42]. The electrodeposition method is accessible for the preparation of magnetic/non-magnetic multilayers in which the magnetic layers are composed of the FM elements Fe, Co and Ni and their mutual alloys, whereas the non-magnetic spacer layer is in most cases Cu. The basic elements of multilayer fabrication by electrochemical methods have been described in detail in some recent reviews [28,31,32,33].

The easiest way of producing magnetic/non-magnetic multilayers by electrodeposition is the application of the two-pulse plating technique from a single bath containing ions of both the magnetic and non-magnetic components [28,33]. A high-current/high-potential pulse serves for the deposition of the magnetic layer and a low-current/low-potential pulse for the deposition of the non-magnetic layer. The layer thicknesses are controlled by the length of the deposition pulses. From the set pulse length and the measured current flowing through the electrochemical cell, the total charge during the pulse can be calculated, which can then be converted into the layer thickness via Faraday’s law. Whereas the deposition of a pure non-magnetic layer can be assured with the single-bath technique, the incorporation of a small amount of the non-magnetic element into the magnetic layers is unavoidable. However, by an appropriate choice of the bath formulation (much higher concentration of the ions of the magnetic elements in comparison with the ionic concentration of the non-magnetic metal) and the deposition conditions [28], this contamination can be minimized to a few atomic percent which does not influence the occurrence of the GMR phenomenon in these multilayers.

It should be pointed out, however, that in the majority of papers reported on the GMR of electrodeposited multilayers [27,28,29,30,31,32,33], two major aspects of the deposition conditions and the magnetoresistance evaluation have not been properly considered, although these aspects have a profound influence on the interpretation of the GMR results obtained.

First, in most cases, the deposition conditions of the magnetic and non-magnetic layers did not ensure that the actual layer thicknesses corresponded to the nominal layer thicknesses set during deposition by using Faraday’s law. It was demonstrated [43] that this is only ensured if the non-magnetic layer is deposited at an electrochemically-optimized potential (which also ensures, at the same time, that no magnetic atoms are codeposited into the non-magnetic layer since this would also be deleterious for the GMR effect). It was shown [44,45] that, in the case of Co/Cu multilayers, instead of a targeted Co(3.4 nm)/Cu(2.0 nm) multilayer, the actual thicknesses can be as different as Co(2.0 nm)/Cu(3.4 nm) due to an improperly chosen (not sufficiently negative) Cu deposition potential (in case the Cu layer is deposited under galvanostatic control with definitely lower rate than the diffusion-limited current density, such layer thickness changes always occur [28]). With the exception of a few cases [46,47,48,49,50,51,52], the investigation of the spacer layer thickness dependence of the GMR has not been carried out on multilayers prepared by electrochemically-optimized Cu deposition potential. Therefore, the latter reported GMR data do not represent a dependence on the true spacer layer thickness.

Second, an often-overlooked problem is that the magnetic layers of electrodeposited multilayers are not always purely ferromagnetic, but may also contain superparamagnetic (SPM) regions magnetically decoupled from the ferromagnetic regions [28,53]. The presence of such SPM regions can be recognized from the non-saturating behavior of the MR vs. magnetic field data, namely, saturation is often not reached up to magnetic fields as high as *H* = 10 kOe [25,26]. In such cases, the GMR was found to consist of two contributions [28,53]: a GMR_FM_ term arising from scattering events of electrons traveling through the spacer layer between two ferromagnetic regions and a GMR_SPM_ term arising from scattering events of electrons traveling through the spacer layer between a ferromagnetic and an SPM region in either direction (the contribution of the also possible electron pathways between two SPM regions was found to be generally negligible). However, when looking for an oscillatory GMR behavior as a function of the spacer layer thickness, most studies have completely neglected the separation of the often non-negligible GMR_SPM_ term from the measured total GMR, whereas one should expect an oscillatory behavior evidently for the GMR_FM_ term only. It should be noted that not only electrodeposited multilayers are prone to the occurrence of a GMR_SPM_ term, but it can also occur in sputtered multilayers, e.g., on rough substrates [54].

### 1.3. Spacer Layer Thickness Dependence of the GMR in Electrodeposited Multilayers: Current Status and Open Questions

In the present paper, with new experimental results on Ni-Co/Cu multilayers, our main focus is on the dependence of the GMR magnitude on spacer layer thickness in electrodeposited multilayers. As pointed out in the previous subsection, this requires that the spacer layer should be deposited at the electrochemically-optimized deposition potential and that the GMR_FM_ term should be extracted from the experimental data if there is also a GMR_SPM_ term in the measured magnetoresistance. These conditions were simultaneously fulfilled only for some previous reports on Co/Cu [46,47,48,49,50], Co-Fe [51] and Ni-Co/Cu [52]. All of these studies demonstrate the absence of an oscillatory GMR as a function of the spacer layer thickness, whereas there are numerous reports on the observation of an oscillatory GMR in various electrodeposited multilayers (for detailed references, see, e.g., Ref. [28]). In Section 6, we will point out, with a focus on the Ni-Co/Cu multilayers, that the conclusions for oscillatory GMR behavior are incorrect and resulted from improper sample preparation conditions and/or from neglecting the superparamagnetic contribution to the observed magnetoresistance.

The present work on Ni-Co/Cu multilayers strongly parallels our previous study on Co/Cu multilayers [47], in which we revealed the origin of the absence of oscillatory GMR behavior. It was demonstrated, with magnetic and MR measurements, that the reason is the absence of an AF coupling between adjacent magnetic layers. Instead, an FM coupling exists at low spacer layer thicknesses due to pinholes in the spacer layer whereas for larger spacer thicknesses the magnetic layers become completely uncoupled. In an uncoupled state, the GMR arises from the random alignment of layer magnetizations which provides an immediate explanation for the much lower GMR in the electrodeposited multilayers [28] in comparison with the physically deposited multilayers with strong AF coupling.

Although our previous report [52] on the spacer layer thickness dependence of GMR in Ni-Co/Cu multilayers already revealed the lack of an oscillatory GMR in this system, the results of the current magnetic and MR measurements only confirm that the same picture emerges for the Ni-Co/Cu multilayers as was deduced for the Co/Cu multilayers [47]. Furthermore, the present study is complemented with such an extended structural investigation by X-ray diffraction which was not performed in our previous work on Ni-Co/Cu multilayers [52]. This new structural information confirmed that the present Ni-Co/Cu multilayers were constituting a highly coherent superlattice stacking sequence.

The high structural quality of the present samples enabled us to assess the nucleation and growth of the spacer layer, which is a key factor in the evolution of GMR with spacer layer thickness. We will particularly discuss this feature and the difference in the layer formation between physically deposited and electrodeposited multilayers. A comparison will be made between the electrodeposited Co/Cu and Ni-Co/Cu multilayers from the viewpoint of the transition from the ferromagnetically coupled state to the uncoupled state upon increasing the spacer layer thickness. The eventual influence of substrate and its roughness on layer formation will also be considered.

The major purpose of the present work was, therefore, to investigate the spacer layer thickness dependence of GMR for a wider spacer thickness range than previously studied [52], in electrodeposited Ni-Co/Cu multilayers prepared with an electrochemically optimized Cu deposition potential. A Ni-rich magnetic layer was chosen in our work as the results of Bian et al. [55] on sputtered Ni-Co/Cu multilayers in this composition range indicated a relatively large GMR with a low saturation field, i.e., high sensitivity. 

Along this line, a series of electrodeposited Ni-Co/Cu multilayers were prepared with spacer layer thicknesses ranging between 0.5 nm and 7 nm, whereas the magnetic layer thickness and the total multilayer thickness remained constant. Sample preparation and structural characterization details, as well as the MR and magnetic measurement methods, will be described in Section 2. The results of the structural studies will be presented in Section 3, whereas the magnetic properties and the MR characteristics will be presented in Section 4 and Section 5, respectively. Section 6 will be devoted to discussing a comparison with relevant previous results on Ni-Co/Cu and Co/Cu multilayers. Section 7 will provide a summary of the present work.

## 2. Experimental

### 2.1. Sample Preparation and Composition Analysis

The Ni-Co/Cu multilayers were electrodeposited from an aqueous electrolyte prepared with some modifications of the bath used to obtain Ni-Co/Cu multilayered nanowires [56]. The final bath composition was 0.7092 mol/L NiSO_4_, 0.0308 mol/L CoSO_4_, 0.01 mol/L CuSO_4_, 0.1 mol/L Na_2_SO_4_, 0.25 mol/L H_3_BO_3_, 0.25 mL/L HSO_3_NH_2_.

All the samples were deposited on a [100]-oriented, 0.26 mm thick Si wafer covered with a 5 nm Cr and a 20 nm Cu layer by evaporation. The purpose of the chromium layer was to ensure adhesion and the Cu layer was used to provide an appropriate electrical conductivity for the cathode surface. Electrodeposition was carried out in a tubular cell of 8 mm × 20 mm cross-section, at room temperature, with an upward-facing substrate placed at the bottom of a recessed cell [28,44,52]. This arrangement ensures a lateral homogeneity of the deposits and helps avoid edge effects.

Based on our experience of studying the initial growth stages of electrodeposited Co/Cu multilayers [57], the electrodeposition process always began with the deposition of a 2.5-nm-thick Cu layer on the Si/Cr/Cu substrate. One aim of depositing such an initial Cu layer is to eradicate, at least partially, the influence of the native oxide of the evaporated Cu layer before the deposition of the first Co layer. The other beneficial effect is the reduction in the Cu content in the first Ni-Co layer due to a depletion of the electrolyte at the cathode surface before the first Ni-Co layer deposition, hence minimizing the difference between the first and upcoming magnetic layers. The magnetic Ni-Co layer thickness was kept constant at 2 nm and the total multilayer thickness was approximately 500 nm for each multilayer. 

Accordingly, the electrodeposited Ni-Co/Cu multilayers investigated here had the general structure of Si/Cr/Cu//Cu(2.5 nm)/[Ni-Co(2 nm)/Cu(*t*_Cu_)] × *N* with 0.5 nm ≤ *t*_Cu_ ≤ 7 nm and with a bilayer number *N* between 56 and 200. The double slash symbol (//) refers to the transition between physically deposited and electrodeposited layers. The nanometric multilayer structure of interest is included between the square brackets []. As the multilayer deposition sequence ends with a Cu layer, the latter served simultaneously as a protective surface layer. Altogether, 17 samples of Ni-Co/Cu multilayers with varying Cu layer thicknesses were obtained, on which the MR measurements could be carried out. It was a strategy not to monotonically increase the Cu layer thickness from one sample to another but to follow a random sequence in order to avoid the effect of possible systematic changes in the deposition conditions. The sequence applied was as follows: sample 11 (*t*_Cu_ = 5.0 nm), 14 (4.0 nm), 15 (3.0 nm), 16 (2.0 nm), 17 (1.0 nm), 18 (4.5 nm), 19 (3.5 nm), 20 (2.5 nm), 21 (1.0 nm), 22 (1.5 nm), 23 (0.5 nm), 24 (1.72 nm), 25 (3.25 nm), 26 (3.75 nm), 27 (5.5 nm) 28 (6.0 nm) and 29 (7.0 nm). As we can see, both samples 17 and 21 were prepared with 1 nm Cu layer thickness for checking reproducibility. (It is noted that samples with serial number less than 11 were prepared for a preliminary optimization of the deposition conditions).

The electrodeposited multilayered structures were prepared using a galvanostatic/potentiostatic (G/P) deposition pulse combination [28,44], in which the magnetic layer is deposited by controlling the deposition current (G mode), whereas the non-magnetic layer (pure Cu) is deposited by controlling the deposition potential (P mode). The deposition of the magnetic layer was conducted at a current density of −70 mA/cm^2^. For the Cu layer deposition, the deposition potential was maintained at −585 mV with respect to a saturated calomel electrode (SCE). This electrochemically optimized potential for Cu [28,43] was used to ensure that neither a dissolution of the Co layer, nor a Co incorporation into the Cu layer, can occur. At the same time, this condition also ensures that the nominal layer thicknesses calculated by Faraday’s law for both the magnetic and non-magnetic layers agree well with the actual values. In the calculations of the nominal layer thicknesses, a current efficiency of 100% was assumed. This is generally accepted for Cu deposition carried out at the limiting current. For the Ni-Co deposition, the current efficiency was estimated to be approximately 96% in our previous work [58]. The above optimization approach applied for the layer deposition procedure was verified by an X-ray diffraction (XRD) study on electrodeposited Co/Cu multilayers [59,60], in which a good agreement (within about 10%) of the nominal and actual layer thicknesses was obtained. In these latter investigations, the superlattice satellites of the experimental XRD patterns were fitted by XRD patterns, calculated for periodic multilayers consisting of face-centered cubic (fcc) Co and fcc-Cu layers, by using a microstructure model in which the free parameters were the interplanar spacings of the lattice planes in the respective fcc structures, as well as the thicknesses of the Co and Cu layers.

The overall multilayer composition was determined in a TESCAN MIRA3 scanning electron microscope (SEM) equipped with an EDAX Element energy-dispersive X-ray spectrometry (EDS) analyzer. After finalizing the deposition bath composition, multilayer sample 11 with layer thicknesses Ni-Co(2 nm)/Cu(5 nm) was subjected to a detailed composition analysis. Three spots on the multilayer were analyzed and at these spots, the Co content of the magnetic layer with respect to Ni was found to be 35.14, 34.46 and 34.84 at.% Co, the average of which is 34.81 at.% Co. By considering the accuracy of the EDS analysis, the magnetic layer composition can be considered as Ni_65_Co_35_. According to our previous detailed study [61] of electrodeposited Co/Cu multilayers deposited under very similar conditions as the present Ni-Co/Cu multilayers, the Cu content of the magnetic layers was found to be below 1 at.% and this should also be expected for our Ni-Co/Cu multilayers. Nevertheless, for the analyzed Ni-Co/Cu multilayer (sample 11), the overall Cu content defined as the ratio c(Cu)/[c(Cu + c(Ni) + c(Co)] was found to be about 70 at.% Cu. This value corresponds approximately to the expected Cu content of the multilayer estimated based on the nominal layer thicknesses. Since all the multilayers were prepared under the same conditions and from the same bath, the magnetic layer composition Ni_65_Co_35_ should also be the same throughout the whole Ni-Co/Cu series. This is based on our experience [52], according to which the Ni:Co atomic ratio in the magnetic layer is determined mainly by the ionic ratio of Ni and Co in the bath.

### 2.2. XRD Studies

The structure of the Ni-Co/Cu multilayers was studied by XRD [62], using a powder diffractometer (type: Smartlab, manufacturer: Rigaku, Tokyo, Japan) with Bragg–Brentano geometry and a D/Tex detector (applying CuKα radiation with a wavelength of λ = 0.15418 nm). The applied voltage and current were 40 kV and 30 mA, respectively. The step size in the measurement of the diffraction angle (2*θ*) was 0.02°. All the multilayers exhibited a coherent fcc superlattice and the lattice constant was determined from the fcc(111) peak using the Bragg equation. For all samples investigated with XRD, faint shoulders appeared on both sides of the main (111) Bragg reflection, which were identified as corresponding to multilayer satellites. The multilayer periodicity or bilayer repeat length (*Λ*) was obtained from the Bragg angles of the two satellite peaks (2*θ*^−^ and 2*θ*^+^) around the fcc(111) reflection using the following equation: *Λ* = *λ*/[sin*θ*^−^ − sin*θ*^+^] [28].

Based on the broadening of the XRD peaks, the crystallite size was first determined by the Scherrer method [62]. As the physical broadening of the profiles is caused by both the size and strain effects, the Williamson-Hall (W-H) method [63] was also applied, which enabled the separation of the two effects. Due to the anisotropic (i.e., *hkl* dependent) broadening of the XRD peaks caused by lattice defects such as dislocations and stacking faults, the crystallite size was estimated by analyzing the breadth of a harmonic reflection pair. Thus, for the present fcc multilayers, the full width at half maximum (denoted as FWHM with the unit of 1/nm) of peaks (111) and (222) were plotted as a function of the magnitude of the diffraction vector (denoted as *g* with the unit of 1/nm). Then, a straight line was fitted to the two points on the Williamson-Hall plot and the reciprocal of the intercept of this line with the vertical axis yielded an estimate of the crystallite size. The microstrain was derived from the slope of the W-H plot. It is noted that the error of the crystallite size from the Scherrer method is approximately 10%, whereas, from the W-H analysis, it is 15% and the same error applies for the microstrain.

### 2.3. Measurement of Magnetoresistance and Magnetic Properties

The magnetotransport parameters were determined at room temperature by using a four-point-in-line resistance probe. The shunting effect due to the substrate layers was estimated to be negligible for the multilayers investigated here. To obtain the magnetoresistance (*MR*), the resistance was measured as a function of the external magnetic field (*H*) up to ~8 kOe. The MR ratio was defined with the formula *MR*(*H*) = [*R*(*H*) − *R*_0_]/*R*_0_ where *R*_0_ is the resistance maximum/minimum of the sample in a magnetic field close to zero and *R*(*H*) is the resistance in an external magnetic field *H*. The magnetoresistance data were determined in the field-in-plane/current-in-plane geometry in both the longitudinal (*LMR*, magnetic field parallel to the current) and the transverse (*TMR*, field perpendicular to the current) configurations. If one takes the difference between the longitudinal and the transverse component, the anisotropic magnetoresistance (*AMR*) can be obtained: *AMR* = *LMR* − *TMR*.

Furthermore, magnetic measurements at room temperature were also performed for the multilayers while being on their substrates with a vibrating sample magnetometer (VSM) in magnetic fields up to *H* = 8 kOe. Before measuring the *M*(*H*) curves in a selected field range, a magnetic field of *H* = 8 kOe was first used to saturate the samples in their plane. The whole sample, together with its substrate with a lateral size of 8 mm × 20 mm, was inserted into the VSM. The large rectangular sample was attached to the sample holder, symmetrically, and with its long axis being horizontal. The sample holder was placed at the same vertical position in the VSM as for the measurement of the usual sample sizes (5 mm × 5 mm). Although in such a case most of the sample is out of the homogeneity range of the detection coil, we have demonstrated in a previous paper [38] that the *M*(*H*) curves are practically identical for the small and large sample sizes.

## 3. Structural Studies by XRD

### 3.1. XRD Patterns and Satellite Reflections

The XRD studies were carried out for the Ni-Co/Cu multilayers with spacer layer thicknesses *t*_Cu_ = 1 nm (sample 17), 1.5 nm (sample 22), 1.72 nm (sample 24), 2.0 nm (sample 16), 3.0 nm (sample 15), 4.5 nm (sample 18), 5.0 nm (sample 11), 5.5 nm (sample 27) and 7.0 nm (sample 29). The XRD patterns indicated that an fcc superlattice structure was formed [64,65], as at each visible reflection, only a single line was observed, the position of which was intermediate between the corresponding reflections of the fcc phase of the constituent metals Ni, Co and Cu. A strong (111) texture was obtained for each multilayer investigated. All the measured XRD patterns revealed multilayer satellite peaks around the Bragg reflections fcc(111), fcc(200) and fcc(311), but the satellites around the fcc(220) reflection could not be resolved due to the large substrate Si peak at approximately 70 degrees. The presence of satellites indicates a good coherent growth of the multilayer stacks [64,65] in our samples.

**Figure 1:**
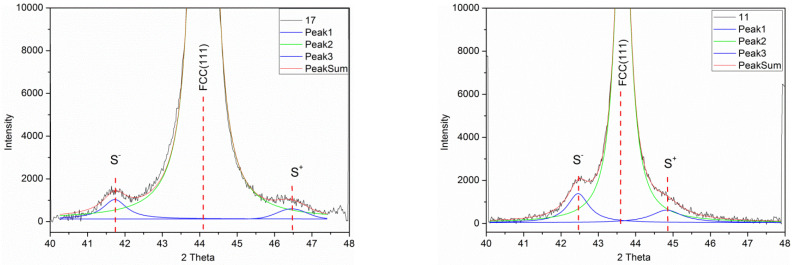
XRD pattern (black line) around the main fcc(111) reflection for the Ni-Co/Cu multilayers with *t*_Cu_ = 1 nm (sample 17, **left panel**) and *t*_Cu_ = 5.0 nm (sample 11, **right panel**) spacer layers. The green line represents the fit for the main reflection (peak 2), and the blue lines represent the fits for the lower-angle satellite peak S^−^ (peak 1) and the higher-angle satellite peak S^+^ (peak 2). The red line is the sum of the three fitted peaks. From the satellite peak positions the bilayer length was obtained as *Λ*_XRD_ = 4.0 nm for sample 17 (*Λ*_nom_ = 3.0 nm) and *Λ*_XRD_ = 8.0 nm for sample 11 (*Λ*_nom_ = 7.0 nm).

The XRD patterns around the main (111) reflection are shown for two multilayers with different spacer layer thicknesses in Figure 1, together with the fits for the main peak and for the two satellite peaks. The red line is the sum of the three fitted peaks and it matches the experimental data (black line) very well. Lorentzian functions were used for fitting the main peak and the satellite peaks. Very similar results were obtained for the other Ni-Co/Cu multilayers. From the satellite peak positions, the bilayer period *Λ*_XRD_ can be deduced, as given in Section 2.2.

**Figure 2:**
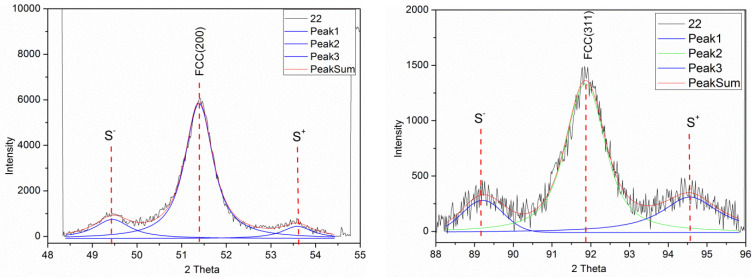
XRD pattern (black line) around the fcc(200) reflection (left panel) and around the fcc(311) reflection (right panel) for the Ni-Co/Cu multilayer with *t*_Cu_ = 1.5 nm thick spacer layer (sample 22). The green line represents the fit for the main reflection (peak 2), and the blue lines represent the fits for the lower-angle satellite peak S^−^ (peak 1) and the higher-angle satellite peak S^+^ (peak 2). The red line is the sum of the three fitted peaks. From the satellite peak positions, the bilayer length was obtained as *Λ*_XRD_ = 4.7 nm for the (200) reflection and *Λ*_XRD_ = 4.6 nm for the (311) reflection. The satellites around the main reflection (111) yielded *Λ*_XRD_ = 4.5 nm whereas the nominal bilayer length was *Λ*_nom_ = 3.5 nm.

Figure 2 shows the XRD patterns for the Ni-Co/Cu multilayer with *t*_Cu_ = 1.5 nm thick spacer layer (sample 22) for the higher-order reflections (200) and (311) which also clearly reveal the presence of satellite peaks. Although the intensity is low and the noise is larger than for the main (111) reflection, a reliable fit of the peaks could be carried out. As indicated in the figure caption, the *Λ*_XRD_ values deduced from these fits of the satellites at these higher-order reflections were in good agreement with the corresponding value obtained from the main (111) reflection. The higher-order reflections could be similarly evaluated for all the other multilayers except for the very weak satellites around the (200) reflections of sample 17 with the thinnest spacer layer (*t*_Cu_ = 1 nm).

**Figure 3:**
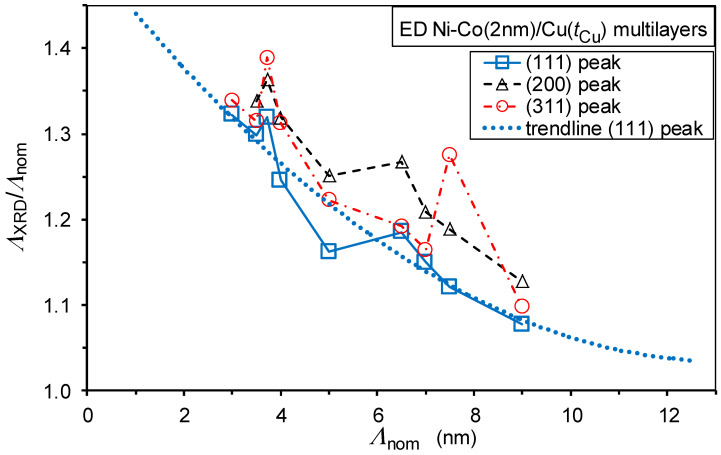
Ratio of the nominal and measured bilayer periods, *Λ*_XRD_/*Λ*_nom_ vs. nominal bilayer thickness *Λ*_nom_ for the present Ni-Co/Cu multilayers as derived from satellites around three Bragg reflections as indicated in the legend. The dotted line is just for indicating the overall trend for the data derived from the satellites around the (111) reflection.

The bilayer period data, derived from the XRD satellite peak positions around the main (111) and the two higher-order reflections (200) and (311) for the present Ni-Co/Cu multilayers, are displayed in Figure 3, in the form of a *Λ*_XRD_/*Λ*_nom_ vs. *Λ*_nom_ plot. By considering the low intensity of the satellites around the higher-order reflections, the agreement is fairly good, at least all three datasets follow the same trend.

It can first be established that *Λ*_XRD_ > *Λ*_nom_ for all the present Ni-Co/Cu multilayers, as generally observed in previous studies on electrodeposited multilayers [45,48,51,52,59,60,61,66]. At the same time, the present data also show a clear trend in that the difference between *Λ*_XRD_, and *Λ*_nom_ becomes smaller and smaller with the increase in the bilayer thickness. It should be noted that, even by taking into account the lower actual current efficiency for the magnetic layers (96% instead of 100% as mentioned in Section 2.1), the nominal bilayer thickness reduces by 3% only for *t*_Cu_ = 1 nm (and only by 1% for *t*_Cu_ = 1 nm). This is much smaller than the experimental *Λ*_XRD_/*Λ*_nom_ ratios, and affects the observed trend to a negligible extent only.

**Figure 4:**
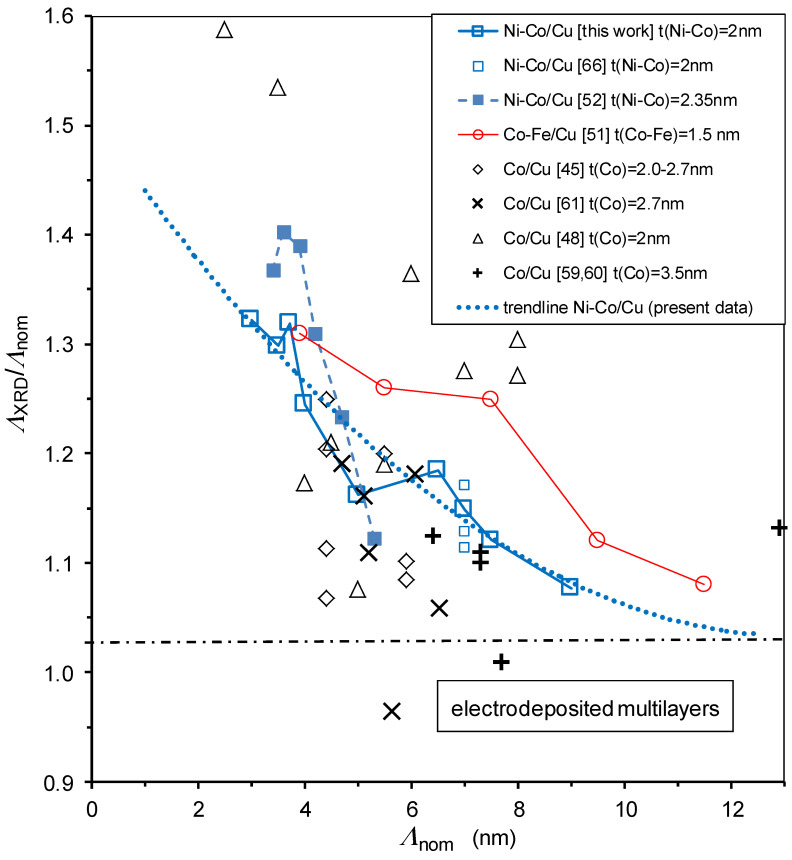
Ratio of the nominal and measured bilayer periods, *Λ*_XRD_/*Λ*_nom_ vs. nominal bilayer thickness *Λ*_nom_ for the present Ni-Co/Cu multilayers (thick squares) and for other electrodeposited multilayers as indicated in the legend. The dotted line is just for indicating the overall trend.

In Figure 4, we have collected similar data from our previous works on electrodeposited multilayers, some of which reveal the same trend (see the full squares [52] and the open circles [51]), but most of the data displayed in Figure 4 can be considered as matching the same overall trend. Although the origin of the observed trend is not yet clear, it is noted that all the data displayed in Figure 4 stem from our previous works and this implies that the preparation conditions of the investigated Ni-Co/Cu, Co-Fe/Cu and Co/Cu multilayers are identical; this may justify why they show very similar behavior. Nevertheless, we are not aware of similar datasets of *Λ*_XRD_/*Λ*_nom_ vs. *Λ*_nom_ for multilayers produced by physical deposition methods, so we cannot ascertain if the above-noticed observation of having *Λ*_XRD_/*Λ*_nom_ > 1 and the approach of this ratio to one towards large bilayer periods are characteristic for electrodeposited multilayers only or if they are a general rule for any multilayer. We shall come back to these issues at the end of this subsection.

**Figure 5:**
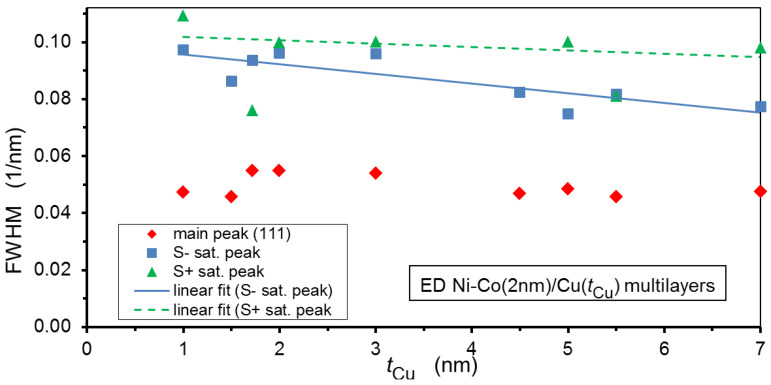
Full width at half maximum (FWHM) for the main (111) peak and the two satellite peaks around it for the present Ni-Co/Cu multilayers. The solid and dashed lines represent a linear fit to the S^−^ and S^+^ peak data, respectively.

From the decomposition fit of the XRD patterns, we could separately determine the broadening of the main (111) peak and its associated two satellite peaks. The full width at half maximum (FWHM) values for the present Ni-Co/Cu multilayers are displayed in Figure 5. The broadening of the main peak is due to the finite size of the coherently scattering domains in the direction perpendicular to the multilayer plane and this will be analyzed in a later subsection. Here, we are interested in the broadening of the satellite peaks, which may reflect the fluctuation of the bilayer period by taking into consideration that the bilayer period *Λ*_XRD_ was determined from the peak positions of the satellite reflections. Figure 5 tells us that the satellite FWHM values are approximately twice as large as that of the main XRD peak. Although it is hard to find a direct connection between the broadening of the main peak and that of the satellites, the large width of the satellites may still be indicative of a significant fluctuation of the bilayer period. On the other hand, although the FWHM data for the satellites are rather scattered, particularly for the S^+^ peaks, due to the low intensity of the satellites, we may still observe in Figure 5 a slight decrease in the satellite broadening towards larger spacer layer thicknesses as indicated by the linear fit to the S^−^ and S^+^ peak data. This might mean that the bilayer period fluctuations due to the island-like growth of the Cu layers for small thicknesses are progressively weakened as the sufficiently thick Cu layers become not only continuous, but also more uniform in thickness.

The above-mentioned conclusions may provide some means for an explanation of the observed relation *Λ*_XRD_/*Λ*_nom_ > 1 and the evolution of this ratio with nominal bilayer thickness *Λ*_nom_. Namely, for low values of the spacer thickness, due to the island-like nucleation of the Cu layers on the magnetic layers, there may be a long-wavelength undulation of the layer planes in the multilayer. If such an undulation exists, the actual layer thicknesses, and thus the bilayer repeat period as well, may be somewhat larger on the flanks than the layer thicknesses at the top or bottom of the undulations. This may explain why the ratio *Λ*_XRD_/*Λ*_nom_ is larger than one for low spacer thicknesses and why the fluctuation of the bilayer thickness is also large here. The reduction of the bilayer thickness fluctuation for larger average bilayer thicknesses, as revealed by the data in Figure 5, may also mean that the degree of undulation is also reduced. As a consequence, the average layer thicknesses also approach the nominal layer thicknesses, explaining the continuous decrease of the ratio *Λ*_XRD_/*Λ*_nom_.

### 3.2. Lattice Parameters and Interplanar Distances

**Figure 6:**
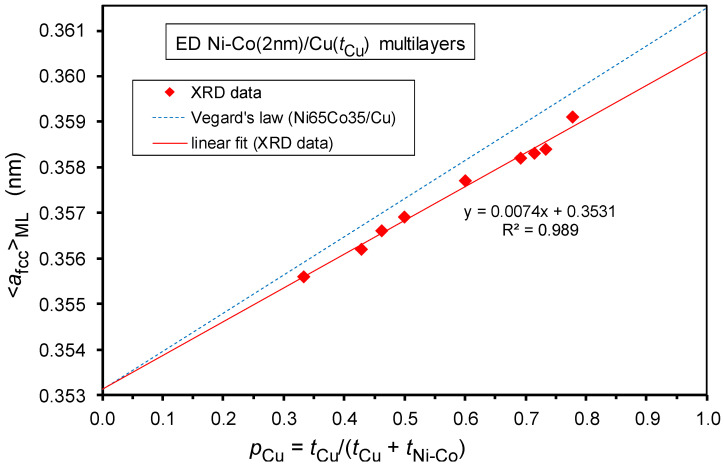
Average lattice parameter <*a*_fcc_>_ML_ in the multilayer stack as determined from the position of the (111) Bragg reflection vs. fractional thickness *p*_Cu_ of the non-magnetic (Cu) layer for the present electrodeposited (ED) Ni_65_Co_35_/Cu multilayers (full diamond symbols). The solid line is a linear fit to the data. The dashed line corresponds to the “multilayer” Vegard’s law for these multilayers [45,67].

As discussed above, a coherent fcc superlattice [64,65] is formed in our Ni-Co/Cu multilayers. From the position of the (111) Bragg reflection, the average lattice parameter <*a*_fcc_>_ML_ was determined. These data are displayed in Figure 6 as a function of the fractional thickness of the spacer layer defined as *p*_Cu_ = *t*_Cu_/(*t*_Cu_ + *t*_Ni-Co_), where *t*_Cu_ and *t*_Ni-Co_ are the thicknesses of the Cu and Ni-Co layers, respectively. Parameter *p*_Cu_ was introduced by Bödeker et al. [67] in order to define an analogy of Vegard’s law for multilayers. One can see that the present <*a*_fcc_>_ML_ data run smoothly along a straight line, as expected for a Vegard’s law type rule. However, there is a definite deviation from a Vegard’s law behavior constructed similarly by fixing the lattice parameter of bulk Cu [68] at *p*_Cu_ = 1 and of a bulk fcc-Ni_65_Co_35_ alloy [69,70] at *p*_Cu_ = 0.

This deviation arises because a real multilayer composed of layers with different lattice parameters are strained. This means that due to the formation of a coherent superlattice (single multilayer reflection peak between the positions of the reflections of the constituent bulk materials), the in-plane lattice plane spacings should match each other in the magnetic (Ni-Co) and the non-magnetic (Cu) layers by the formation of coherent interfaces between the two kinds of layers. The matching constraint implies a lateral compression in the Cu layers (larger equilibrium lattice constant in the equilibrium bulk phase) and a lateral dilation in the Ni-Co layers (smaller bulk equilibrium lattice constant). However, as a consequence of the incompressibility of the metals (Poisson response [67]), the out-of-plane lattice plane spacings should be correspondingly modified in the constituent layers: dilation in the Cu layers and compression in the Ni-Co layers.

**Figure 7:**
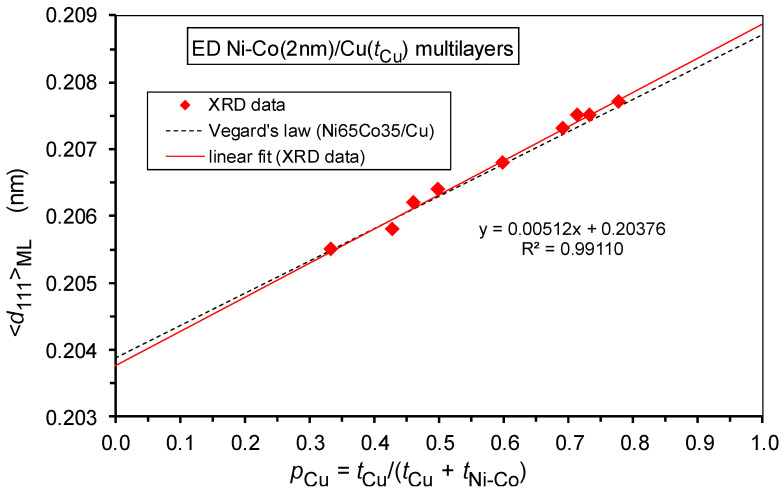
Average lattice spacing <*d*_111_>_ML_ perpendicular to the multilayer plane as determined from the position of the (111) Bragg reflection vs. fractional thickness *p*_Cu_ of the non-magnetic (Cu) layer for the present Ni_65_Co_35_/Cu multilayers (full diamond symbols). The solid line is a linear fit to the data. The dashed line corresponds to the “multilayer” Vegard’s law for these multilayers [45,67].

Furthermore, we should take into account that, due to the geometry of the applied XRD measurement, the position of the fcc(111) reflection corresponds to the out-of-plane lattice plane spacing of the (111) planes. Therefore, not the average lattice parameter <*a*_fcc_>_ML_, but rather the average out-of-plane lattice plane spacing <*d*_111_>_ML_ obtained from the (111) reflection, should be displayed as a function of the fractional Cu layer thickness *p*_Cu_ [45,67]. This is shown in Figure 7 for the present Ni-Co/Cu multilayers and the data are fairly close to the corresponding “multilayer” Vegard’s law [45,67] for <*d*_111_>_ML_, obtained by taking again the bulk values for *p*_Cu_ = 0 and *p*_Cu_ = 1. The agreement is much better than that found for the Co/Cu multilayers [45,67] because the magnetic fcc-Ni_65_Co_35_ alloy is much less prone to the formation of stacking faults as this alloy does not have a (metastable) hcp modification in contrast to pure Co metal. The agreement could have been made even better if, instead of the “non-strained multilayer” Vegard’s law displayed in Figure 7 (dashed line), we had used the “strained multilayer” Vegard’s law (see Equation (7) of Ref. [67] or Equation (2) of Ref. [45]) which takes into account the difference in the elastic constants of Cu and Ni-Co. However, this refinement is already beyond the scope of the present paper and, anyhow, we could only obtain approximate estimates for the necessary elastic constants of the magnetic Ni-Co layer.

### 3.3. Crystallite Size and Microstrain

**Figure 8:**
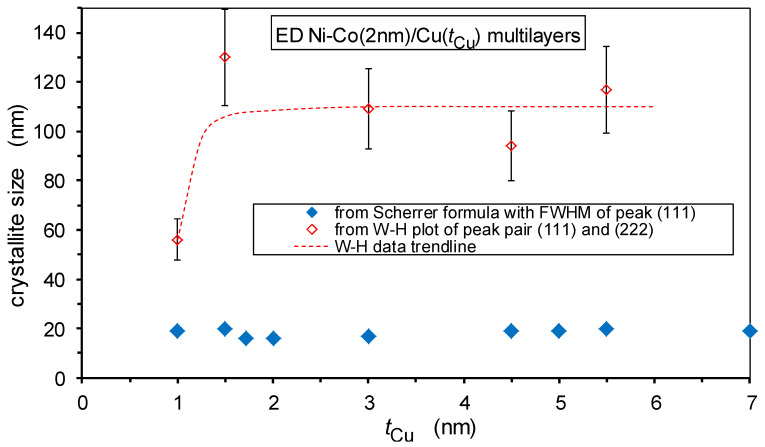
Crystallite size in the growth direction for the present Ni_65_Co_35_/Cu multilayers from the Scherrer formula (full diamond symbols) and from the W-H plots (open diamond symbols; the dashed line is just an estimated trendline).

Important microstructural features can be derived from the broadening of the XRD lines. First, the FWHM value of the main (111) Bragg reflection was used to obtain an estimate of the crystallite size by using the Scherrer formula [62]. Due to the applied XRD measurement geometry, this parameter characterizes the size of the coherently scattering domains along the normal of the multilayer plane, i.e., along the growth direction. These data are displayed in Figure 8 with the full diamond symbols. The crystallite size values derived from the Scherrer formula are scattered between approximately 15 nm and 20 nm for the whole range of Cu layer thicknesses and can be considered constant within error. These values are approximately two to five times larger than the bilayer thicknesses, i.e., they extend over several bilayers.

However, due to the microstrains present in these multilayers, the actual size of the coherently scattering domains is substantially underestimated by the Scherrer formula and analysis through the Williamson–Hall plots are required [63]. These data are shown in Figure 8 by the open diamond symbols. We can see that the crystallite sizes from the W-H plots are significantly larger than the values from the Scherrer formula and extend over a fairly large number of bilayers, particularly above an approximately 1.5 nm Cu layer thickness. It is particularly important to note that between approximately 1 nm and 1.5 nm Cu layer thickness, the out-of-plane direction size of the coherently scattering domains increases suddenly by a factor of two. This implies that beyond a critical Cu layer thickness, a large fraction of the multilayers can be considered to consist of single crystals, with a height of at least 100 nm, which is much larger than a bilayer period. The sudden increase of the vertical crystallite size may be considered as a sign of the formation of a much better defined Cu layer; thus implying a more structurally perfect multilayer beyond this critical Cu layer thickness.

**Figure 9:**
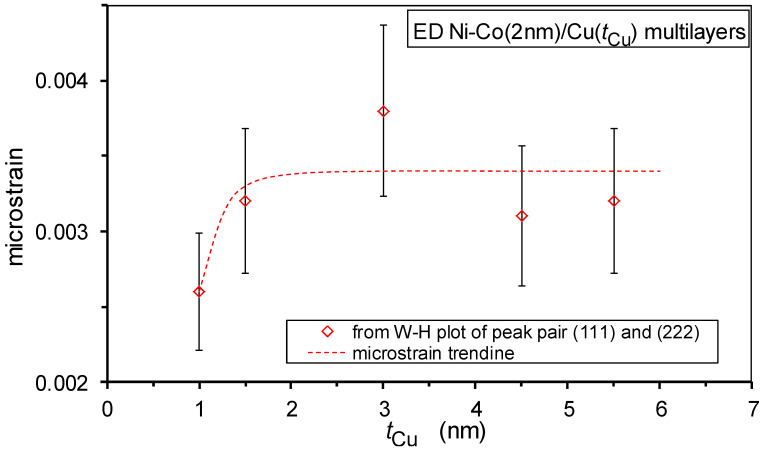
Microstrain for the present Ni_65_Co_35_/Cu multilayers as derived from the W-H plots (open diamond symbols; the dashed line is just an estimated trendline).

From the slope of the W-H plots, the microstrain can be deduced and these data are displayed in Figure 9. A slight increase in the microstrain can be observed beyond a Cu layer thickness of approximately 1 nm. This may be connected with the fact that, as discussed above, in this thickness range, a real superlattice structure with coherent interfaces between the constituent layers is formed which inherently implies larger strains in both layers due to the lattice matching of the two layers with different lattice parameters in their bulk form. It should be noted that the strong crystallographic texture in the nanocrystalline films can yield an apparent narrowing of the first few XRD peaks [71]. Therefore, the crystallite size, determined by either the Scherrer formula or the W-H plot, as well as the microstrain obtained by the W-H plot may be somewhat higher than the real values. However, since all the investigated multilayers exhibited the same (111) texture and we were interested in the evolution of these parameters with spacer layer thickness only, all the above conclusions are not affected by this overestimation of the deduced microstructural parameters.

## 4. Magnetic Properties

The magnetization curves were very similar for all the Ni-Co/Cu multilayers and qualitatively exhibited the same behavior as the Co/Cu multilayers in our previous work [38]. The hysteresis loops were very steep and the magnetization reached saturation in magnetic fields between 1 and 2 kOe. The coercive field *H*_c_ monotonously increased with the spacer layer thickness; this will be discussed in Section 5, together with the corresponding magnetic field *H*_p_ at the maximum of the magnetoresistance curves.

**Figure 10:**
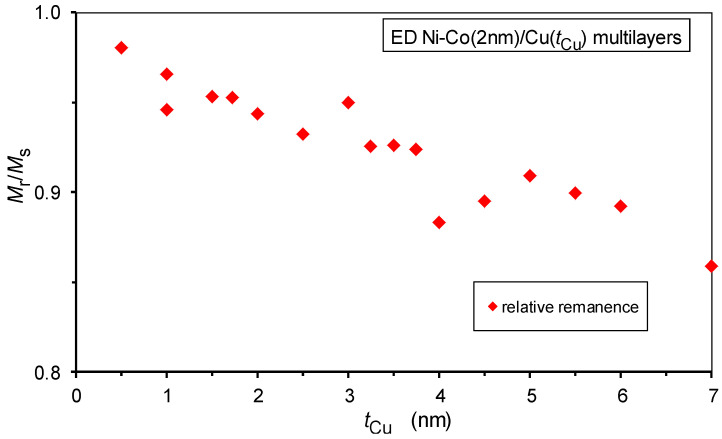
Relative remanence *M*_r_/*M*_s_ vs. non-magnetic layer thickness *t*_Cu_ for the present Ni_65_Co_35_/Cu multilayers as obtained from the magnetic hysteresis loops.

The hysteresis loops exhibited a high relative remanence *M*_r_/*M*_s_ for all multilayers where *M*_s_ is the saturation magnetization and *M*_r_ is the remnant magnetization; the *M*_r_/*M*_s_ data are displayed in Figure 10. It is generally considered [23,72] that the quantity *M*_r_/*M*_s_ of a multilayer gives an estimate of the sample fraction which is not antiparallel aligned (i.e., not antiferromagnetically coupled) at *H* = 0 magnetic field. The relatively high *M*_r_/*M*_s_ values on our Ni-Co/Cu multilayers speak for a rather small AF fraction, if at all. Nevertheless, we can see that the relative remanence slightly decreases with the increasing spacer layer thickness; we will come back to this point later, when discussing the magnetoresistance results.

**Figure 11:**
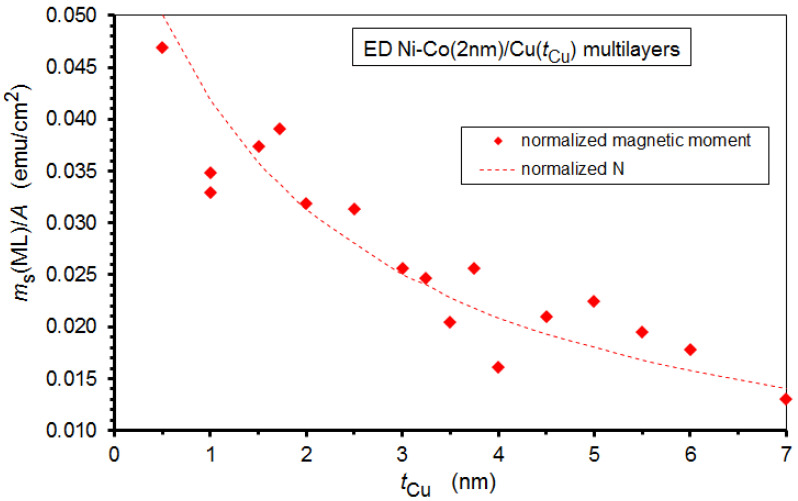
Measured saturation magnetic moment per unit surface area *m*_s_(ML)/*A* (diamond symbols) for the present Ni_65_Co_35_/Cu multilayers with various non-magnetic layer thicknesses (*t*_Cu_). The dashed line represents the appropriately normalized bilayer number to demonstrate that the measured magnetic moment is reduced just in the same manner as the decrease of the number of magnetic layers in the multilayer stack.

To compare the measured saturation magnetic moment *m*_s_(ML) for the various Ni-Co/Cu multilayer samples, we applied the following procedure. Since the magnetic measurements were performed on the samples while being on their substrates, the mass of the multilayers could not be measured separately; thus, their magnetization also could not be determined. Therefore, we proceeded by calculating the normalized magnetic moments of the multilayers. As the individual multilayer samples had a slightly different surface area (*A*), for each multilayer we calculated the magnetic moment per unit surface area *m*_s_(ML)/*A*. The experimental *m*_s_(ML)/*A* values are displayed in Figure 11 and we can see that the data follow a monotonously decreasing trend, with some scatter which probably derives from the inaccuracy of the surface area determination. The decrease is due to the decreasing number of magnetic layers in the multilayer stack with increasing Cu layer thickness because the total multilayer thickness was maintained constant, at around 500 nm for the whole series. When displaying the bilayer number (after an appropriate normalization) by the dashed line in Figure 11, one can see that the reduction of the surface area magnetic moment follows the behavior of the decrease in the number of magnetic layers in the multilayers with increasing spacer thickness.

## 5. Magnetoresistance Results

### 5.1. MR(H) Curves

**Figure 12:**
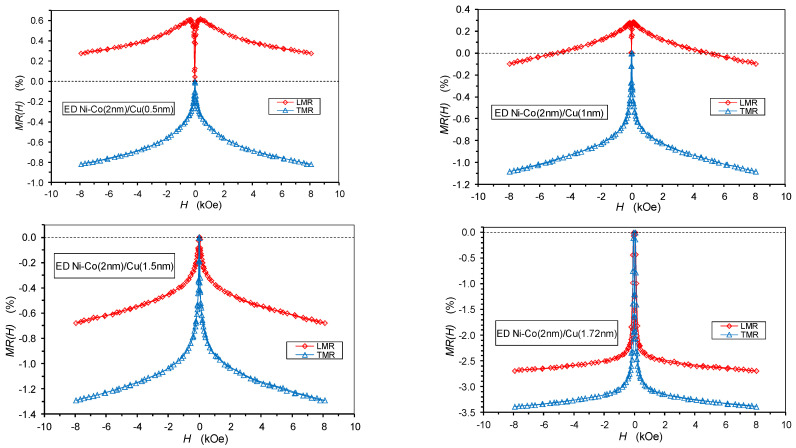
MR(*H*) curves for the four Ni-Co/Cu multilayers with the thinnest Cu layers: *t*_Cu_ = 0.5 nm (**upper left panel**), 1 nm (**upper right panel**), *t*_Cu_ = 1.5 nm (**lower left panel**) and *t*_Cu_ = 1.72 nm (**lower right panel**).

The magnetoresistance results are shown for those four multilayers which have the smallest spacer layer thicknesses (*t*_Cu_ = 0.5 nm, 1 nm, 1.5 nm and 1.72 nm) as these MR(*H*) curves (Figure 12) illustrate well the progressive evolution of the multilayer structure with spacer layer thickness, which then has a profound influence on the magnitude of the GMR effect. It will soon become clear that it is more instructive to start with the Ni-Co/Cu multilayer with *t*_Cu_ = 1.72 nm, for which the MR(*H*) curve is shown in the lower right panel of Figure 12, as all multilayers with *t*_Cu_ > 1.72 nm qualitatively exhibited the same MR(*H*) curves, with the exception of the larger hysteresis of the MR(*H*) curves with the increasing spacer layer thickness.

The MR(*H*) curve of the multilayer with *t*_Cu_ = 1.72 nm reveals a typical GMR multilayer behavior in that (i) both the LMR and TMR components are negative for the whole range of the magnetic fields investigated [28]; (ii) the magnitude of TMR is larger than that of LMR due to the bulk AMR effect of the magnetic layers [5,73,74]; and (iii) a rapid saturation of the magnetoresistance at around 2.5 kOe magnetic field, which is followed by an approximately linear, slight decrease of the resistivity due to the progressive suppression of the magnetization fluctuations (spin waves) with increasing magnetic field [75,76,77]. Therefore, the Ni-Co/Cu multilayer with *t*_Cu_ = 1.72 nm (and all the other multilayers with *t*_Cu_ > 1.72 nm) can be considered to contain magnetic layers with fully ferromagnetic behavior without any noticeable SPM character, i.e., the observed GMR is contributed to by the GMR_FM_ term only.

We should also note that the magnetic-field-induced resistivity change at the highest magnetic fields applied is approximately 3% for the multilayer with *t*_Cu_ = 1.72 nm, whereas it is approximately 1%, or even less, for the Ni-Co/Cu multilayers with thinner spacer layers. This is because, for *t*_Cu_ = 1.72 nm, the spacer layer is already rather uniform in thickness and can provide a sufficient separation of the magnetic layers from each other, thus enabling the development of a significant GMR effect between the adjacent magnetic layers. On the other hand, for multilayers with *t*_Cu_ < 1.72 nm, the spacer layers are probably not yet uniform in thickness and may contain so-called pinholes which are then filled up by Ni and Co atoms during the deposition of the subsequent magnetic layer. Through the pinholes, a direct FM coupling develops between the adjacent magnetic layers and some fraction of the neighboring layer magnetizations will be aligned, parallel to each other in zero magnetic field, reducing the overall GMR effect. This picture can be well followed in Figure 12 by looking at the MR(*H*) curves with increasingly smaller spacer layer thicknesses. At *t*_Cu_ = 0.5 nm and 1 nm, we can even observe that the LMR(*H*) curves start to increase first for very low magnetic fields as is typical for bulk FM metals [5,38,58,73,74,77]. This implies that, in these cases, the spacer layer is certainly discontinuous to the extent that, in a rather large volume fraction of the multilayer, the adjacent magnetic layers coalesce with each other, thus forming large contiguous ferromagnetic regions that exhibit the AMR behavior typical for bulk ferromagnets.

Nevertheless, whereas the AMR effect dominates the observed MR(*H*) curves for spacer layer thicknesses *t*_Cu_ = 0.5 nm, 1 nm and 1.5 nm, even in these Ni-Co/Cu multilayers a small, but clear GMR contribution to the magnetoresistance can be identified, as we will demonstrate in the quantitative analysis in the next section. A GMR contribution can arise here from those regions of the multilayer where, locally, the spacer layer is continuous and, thus, can provide an appropriate separation of the two adjacent layers; then, a non-aligned magnetization orientation of the magnetizations of the two layers can occur in the zero magnetic field. It can be observed that the approach to saturation requires somewhat higher magnetic fields (ca. 4 kOe) for these thinner spacer layers than for *t*_Cu_ > 1.5 nm (2.5 kOe). This fact may arise from the not sufficiently smooth and irregularly layered form of the magnetic layers at some regions and, perhaps, also from the presence of a small amount of SPM regions which also causes an increase of the saturation field.

In summary, we can say that the evolution of the MR(*H*) curves of the present Ni-Co/Cu multilayers in Figure 12 suggests that, for spacer layer thicknesses beyond *t*_Cu_ = 1.5 nm, a well-defined multilayer structure is formed with a dominant GMR effect. This is in accordance with the conclusions drawn from the XRD studies and will be corroborated with further results presented below.

### 5.2. Spacer Layer Thickness Dependence of the GMR and AMR

As noted at the beginning of the previous section, the investigated Ni-Co/Cu multilayers with at least 1.72 nm spacer layer thickness all exhibited MR(*H*) curves similar to that shown in the lower right panel of Figure 12. These multilayers have a dominant GMR effect deriving from spin-dependent scattering events occurring while electrons are traveling between two adjacent magnetic layers via the non-magnetic spacer layer. Due to the high remanence revealed by the magnetic measurements, we may anticipate that there is no AF coupling between the magnetic layers. With the increasing spacer layer thickness, the magnetic layers become progressively uncoupled and, within each layer, they have a random orientation of the magnetization which can even vary from site to site as in zero magnetic field the magnetic layers can break up into magnetic domains within the layer planes [24].

Superimposed on the GMR effect, the splitting of the LMR(*H*) and TMR(*H*) curves speaks for the presence of an AMR effect of bulk ferromagnets, which is due to electron scattering events within the magnetic layers. The AMR effect provides the dominant contribution to the observed magnetoresistance in the multilayers with thin spacer layers (*t*_Cu_ = 1.5 nm and below) besides a minor GMR contribution.

For the quantitative analysis of the MR(*H*) curves, we will proceed as follows: by fitting a straight line for the MR(*H*) data above the saturation field (which was about 2.5 kOe for multilayers with *t*_Cu_ > 1.5 nm and about 4 kOe for thinner spacer layers), we extrapolate the measured magnetoresistance data to *H* = 0. This procedure yields the LMR_s_ and TMR_s_ values where the subscript s refers to the fact that the extrapolation was made from the magnetically saturated region [58,77,78]. The anisotropic magnetoresistance ratio is obtained as AMR = LMR_s_ − TMR_s_ [5,77,78]. In analogy with the isotropic contribution to the zero-field resistivity of ferromagnets [5,78], the isotropic GMR can be obtained as GMR = (LMR_s_ + TMR_s_)/3 (we will take the absolute value of the resistivity change, i.e., we will have GMR > 0).

**Figure 13:**
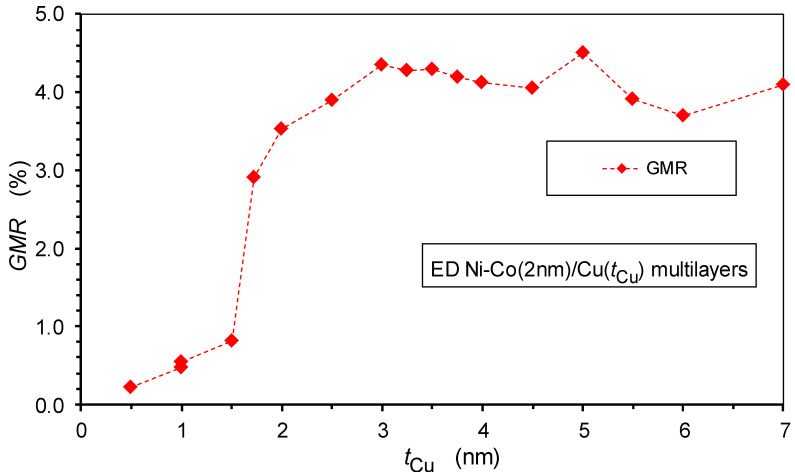
Evolution of the magnitude of the GMR effect with spacer layer thickness *t*_Cu_ for the present Ni_65_Co_35_/Cu multilayers.

The GMR values for the present Ni-Co/Cu multilayers are shown in Figure 13 as a function of the spacer layer thickness. As already indicated by the MR(*H*) curves displayed in Figure 12, there is an abrupt increase in the GMR when the spacer thickness passes 1.5 nm. This is because, in this thickness range, the spacer layer starts to provide a good separation with a sudden drop in the pinhole density, leading to a substantial magnetic decoupling of the adjacent magnetic layers from each other. Further up, to approximately 3 nm spacer thickness, there is still some improvement in the multilayer structure, leading to a slight additional increase in the GMR; subsequently, the GMR remains approximately constant or at most a slight decrease can be observed towards large thicknesses.

We should recall at this point the results of structural studies in Section 3, where multilayer satellites can be observed for all of the multilayers studied by XRD. This implies the presence of a periodic repetition of the magnetic/non-magnetic bilayer units, even for very small spacer layer thicknesses. Indeed, the relative intensity of the satellite reflections was rather small in the latter cases, which is due to the fact that only part of the whole multilayer shows a regular coherent layered structure. In line with this, the GMR magnitude was also found to be rather small, which is again due to the relatively small fraction of the regularly formed layered structure. All of these considerations are further substantiated by the strong increase in the crystallite size in the growth direction (Figure 8) and of the microstrains (Figure 9) in the same spacer layer thickness range as the GMR.

The discontinuous nature of the spacer layer at small thicknesses is a result of the nucleation of the large Cu atoms on the previously deposited Ni-Co magnetic layer, consisting of smaller atoms than Cu, which does not proceed via layer-by-layer growth, but rather with island-like growth. The islands then grow both laterally and vertically on the substrate layer and the Cu layer remains discontinuous until a critical lateral size of the islands. After the islands reach each other and coalesce during the lateral growth, a complete coverage occurs, and it is likely that the pinholes disappear and a continuous, compact Cu layer is formed, which completely separates the adjacent magnetic layers.

**Figure 14:**
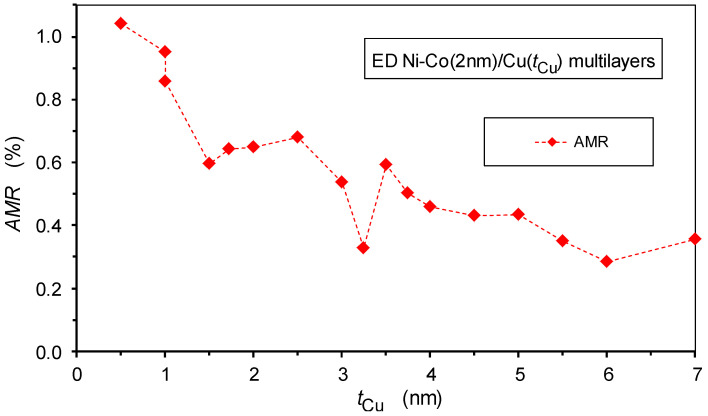
Evolution of the AMR ratio with spacer layer thickness *t*_Cu_ for the present Ni_65_Co_35_/Cu multilayers.

We have also evaluated the AMR ratio for the present Ni-Co/Cu multilayers and the results in Figure 14 show a rather monotonic decrease of AMR with the increasing spacer layer thickness. As the AMR effect occurs inside the magnetic layers only, the AMR ratio should be reduced due to a diminution of the contribution of the magnetic material to the multilayer resistivity, as the number of magnetic layers dropped from 200 to 56 in our sample series.

### 5.3. Spacer Layer Thickness Dependence of the Hysteresis Parameters Hc and Hp

**Figure 15:**
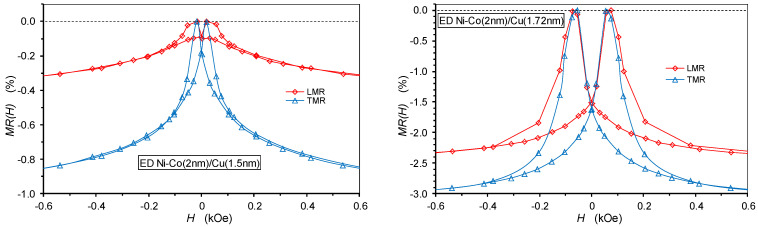
MR(*H*) curves at large resolution for the present Ni_65_Co_35_/Cu multilayers with spacer layer thicknesses *t*_Cu_ = 1.5 nm (**left panel**) and *t*_Cu_ = 1.72 nm (**right panel**).

Similarly to the *M*(*H*) magnetization curves, the *MR*(*H*) curves also exhibit a hysteresis behavior as both sets of data reflect, in some sense, the remagnetization process, which should show a hysteretic behavior in the presence of any kind of magnetic anisotropy. For all the investigated multilayers, the *MR*(*H*) curves exhibited a hysteresis and the low-field MR(*H*) curves of two multilayers from Figure 12 are displayed in Figure 15. We can observe a significant increase in the magnetoresistance hysteresis when increasing the spacer thickness from 1.5 nm to 1.72 nm. In the same thickness range, where an abrupt rise in the GMR occurred (Figure 13).

The hysteretic behavior can be characterized by the coercive field *H*_c_ of the *M*(*H*) curve and by the peak position *H*_p_ of the *MR*(*H*) curve. The two quantities (*H*_c_ and *H*_p_) reflect critical magnetic field points during the remagnetization process where the distribution of the domain magnetization orientations exhibits an extremum. *H*_c_ and *H*_p_ are usually close to each other, although they are not necessarily equal. This is because the conditions for zero magnetization (*H* = *H*_c_) during the magnetization reversal are not identical with the conditions for the occurrence of maximum/minimum resistivity peaks during cycling the external magnetic field.

At *H* = *H*_c_, the field-direction-projected domain magnetization components, aligned parallel and antiparallel to the magnetic field orientation, sum up to zero. At the field position of the LMR minimum, *H*_p_(LMR), the absolute values of the field-direction-projected magnetization components, irrespective of whether they are aligned parallel or antiparallel to the magnetic field orientation, sum up to a minimum value. As the same domain magnetizations can be split into components parallel (LMR) and perpendicular (TMR) to the magnetic field direction (not to the magnetic field orientation), the TMR maximum, *H*_p_(TMR), should appear at the same magnetic field as the LMR minimum. This was demonstrated by the *MR*(*H*) data of a nanocrystalline Ni sample [77], in which the accuracy of the measured resistivity data and the sharp peak of the *MR*(*H*) curves enabled us to observe the validity of the relation *H*_p_(LMR) = *H*_p_(TMR).

**Figure 16:**
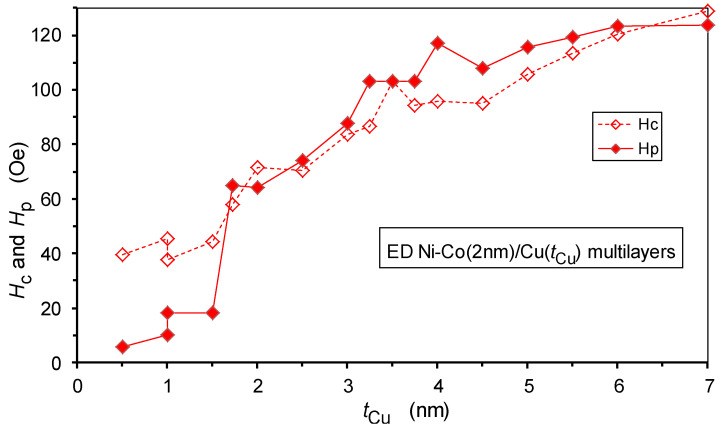
Evolution of the coercive field *H*_c_ and *MR*(*H*) peak position *H*_p_ with spacer layer thickness *t*_Cu_ for the present Ni_65_Co_35_/Cu multilayers.

The variation of the two hysteresis parameters with spacer layer thickness for the present Ni-Co/Cu multilayers is shown in Figure 16. Both *H*_c_ and *H*_p_ have small values for low spacer layer thicknesses, the range for which we have concluded that the spacer layer is partially discontinuous and, therefore, the magnetic layers may have a direct FM coupling. In this sense, the magnetic component of the multilayer structure may be similar to a bulk-like ferromagnet and is expected to exhibit a soft magnetic behavior. On the other hand, the development of a continuous spacer layer beyond approximately *t*_Cu_ = 1.5 nm eliminates the direct FM coupling between the adjacent magnetic layers which then, with the further gradual increase in the spacer thickness, will show a behavior characteristic of individual thin magnetic layers. As a result, with the diminishing FM coupling between the magnetic layers upon the increasing Cu layer thickness, the multilayer coercive field should gradually increase to a value characteristic of individual, uncoupled Co layers with a thickness of approximately 2 nm. It is well known that the *H*_c_ of thin magnetic layers increases roughly proportionally to the inverse of the layer thickness [38,79,80,81]. The data in Figure 16 are in perfect agreement with the picture put forward in Section 5.2 concerning the evolution of the Ni-Co/Cu multilayer structure with spacer layer thickness.

Furthermore, these results are in good agreement with our previous findings [47] on the electrodeposited Co/Cu multilayers prepared under identical conditions. The slight quantitative difference between the saturation value of *H*_c_ and *H*_p_ for the present Ni-Co/Cu multilayers (ca. 120 Oe) and the previously studied [47] Co/Cu multilayers (ca. 90 Oe) complies with the corresponding difference of the magnetic layer thicknesses (2 nm and 2.7 nm, respectively) of the two multilayer systems.

## 6. Discussion: Critical Evaluation of the GMR vs. Spacer Thickness Behavior Reported for Ni-Co/Cu Multilayers

The overall picture emerging from the present magnetoresistance data is that, for electrodeposited Ni_65_Co_35_/Cu multilayers prepared with properly controlling the layer thicknesses through the electrochemically optimized spacer deposition potential, the GMR does not exhibit an oscillatory behavior. Instead, a rather abrupt increase in the GMR occurs beyond a critical Cu layer thickness (ca. *t*_Cu_ > 1.5 nm) and then the GMR remains nearly constant up to the highest spacer thickness (7 nm) investigated here. Supported by the results of XRD and magnetic studies on the same multilayers, the sudden improvement of the GMR in a narrow spacer layer thickness range could be attributed to a change of the Cu layer formation process. This change implies a rather sharp transition from a discontinuous spacer layer for thicknesses up to about 1.5 nm to a smooth and continuous spacer layer, above which the spacer ensures an efficient magnetic decoupling of the adjacent layer magnetizations from each other. It is also important to note that in the lack of an AF coupling, the decoupled state of the magnetic layers leads to a random orientation of layer magnetizations independently of each other. This provides a chance for non-aligned magnetizations of adjacent magnetic layers in some regions, which then results in a GMR effect. Our data indicate that due to the proper control of the deposition of both layers, the observed GMR is of the regular multilayer type, consisting of a GMR_FM_ component only and, for spacer thicknesses above about 1.5 nm, no SPM contribution to the GMR can be identified.

These conclusions will now be compared with previous reports on the spacer layer thickness dependence of the GMR on the widely studied electrodeposited Ni-Co/Cu multilayer system. We will group the reported results according to the observed spacer thickness dependence of the GMR.

### 6.1. Decreasing GMR with Increasing Spacer Thickness

In the very first studies on the observation of the GMR effect in electrodeposited multilayers [25,26], the *MR*(8 kOe) values decreased rapidly with the increase of the nominal spacer thickness from 0.7 nm to 2 nm for the investigated Ni-Co/Cu multilayers; up to 3.5 nm, the GMR remained constant roughly at the same level as in the present Ni-Co/Cu multilayers. It should also be noted that in these studies, the magnetoresistance curves seemed to be far from saturation even at the highest magnetic field applied (8 kOe).

Based on a later developed model [53] for the decomposition of the GMR in electrodeposited multilayers into GMR_FM_ and GMR_SPM_ contributions, we can now establish that the *MR*(*H*) curves reported in Refs. [25,26] were dominated by the GMR_SPM_ term, which is strongly supported by the non-saturating character of the *MR*(*H*) curves. The reason for this behavior is that at the Cu deposition potential of –0.15 V vs. SCE applied in Refs. [25,26], a strong dissolution [43,44] of the previously deposited magnetic layer occurs during the deposition of the Cu layer. As a consequence, the actual magnetic layer thickness will be much smaller than the nominal value (2 nm is this case).

Based on previous results, the expected layer thickness change will now be estimated for the discussed case. It was found for electrodeposited Co/Cu multilayers [44,45] that with the electrolyte concentrations typical for multilayer deposition and at a Cu deposition potential of –0.25 V vs. SCE, the magnetic layer thickness reduces by approximately 1.4 nm (and, correspondingly, the Cu layer increases by the same amount) with respect to the nominal value. The presence of Ni in the magnetic layer reduces the dissolution rate. For example, for Ni-Co/Cu multilayers with a Ni:Co ratio of approximately 50:50 [66], which were prepared by the same Cu deposition potential as the Co/Cu multilayers, the magnetic layer thickness reduction was approximately 0.3 nm. By considering that the Ni-Co/Cu multilayers of Refs. [25,26] had a Ni:Co ratio of about 21:79, we can estimate a reduction in the magnetic layer thickness by approximately 1 nm, i.e., from the nominal 2 nm down to about 1 nm. As the Cu deposition potential applied was more positive in Refs. [25,26] (–0.15 V vs. SCE), the thickness reduction was even larger. As the dissolution is not a uniform process over the whole surface, the strongly dissolved magnetic layer disintegrates at such a low average thickness into small magnetic regions, mostly with SPM behavior, which are embedded in Cu. This clearly explains the shape of the observed *MR*(*H*) curves exhibiting a strong SPM character. Therefore, the spacer thickness dependence of GMR in Refs. [25,26] is not expected to exhibit any oscillatory behavior as only the GMR_FM_ term can show this feature. Furthermore, due to the charge control of the layer thickness, the charge of the dissolution of magnetic atoms should be compensated for by the deposition of excess Cu atoms in the same amount as the dissolved magnetic atoms. Therefore, the spacer thickness dependence should be shifted, upward to at least by 1 nm, as the actual Cu layer thicknesses are larger by roughly this amount than the nominal values.

It follows from the above discussion that if the Cu deposition potential is much more positive than the optimum value, the formation of SPM regions is particularly enhanced when the nominal magnetic layer thickness is as small as 1 nm. Furthermore, strong SPM region formation was observed even for an optimized Cu deposition potential in the case of the Co(1.1 nm)/Cu(1.1 nm) multilayers, i.e., when both layer thicknesses were very small [82]. This is due to the island-like nucleation of the Cu layer on top of the layers of the smaller magnetic atoms, as a result of which the Cu layer becomes discontinuous at low average thicknesses. Combined with the too positive Cu deposition potential, leading to a dissolution of the magnetic layer, the discontinuous Cu layer effect (for *t*_Cu_ = 0.7 nm nominal thickness) can then cause SPM-type *MR*(*H*) behavior to occur even for nominal magnetic layer thicknesses as high as 3 nm [83].

On the other hand, even a too positive Cu deposition potential (–0.3 V vs. SCE) can yield multilayers with a dominating GMR_FM_ term if both nominal layer thicknesses are as high as, e.g., 5 nm [84]. Of course, for the non-optimized, too positive Cu deposition potentials, the reported nominal Cu layer thicknesses are strongly underestimated, by a value of the order of 1 nm, with respect to the actual values, so the reported spacer layer thickness dependences should be considered with this notion [83]. The same concerns about uncertainties in the actual layer thicknesses always arise for cases where the non-magnetic layer was deposited under galvanostatic control [85,86] and where the actual Cu deposition current density has to be kept at a value lower than the diffusion-limited current density.

Following this discussion, we can now return to finding an explanation for the observed initial reduction in the GMR with the increasing spacer layer thickness in Refs. [25,26]. First, we recall that all the nominal Cu layer thickness values should be increased by approximately 1 nm due to the applied too positive Cu deposition potential. Second, due to the strong dissolution of the magnetic layer under these conditions, the specified nominal magnetic layer thickness of 2 nm is reduced down to approximately 1 nm and the remaining magnetic layers primarily consist of SPM regions which are embedded in a Cu matrix. Therefore, the observed large GMR at the initial low spacer thicknesses is due to the granular type GMR (GMR_SPM_ [28,53]), also evidenced by the shape of the *MR*(*H*) curves. With the increasing Cu layer thickness, the zones of SPM regions embedded in Cu are separated by thicker Cu layers, so the fraction of material with an MR contribution continuously reduces, leading to a diminished GMR.

### 6.2. Critical Assessment of Previous Papers Claiming to Have Observed Oscillatory GMR

We should mention here the results of Hua et al. [87,88], who reported a small GMR peak at approximately *t*_Cu_ = 1 nm and a much larger GMR peak at a nominal layer thicknesses of approximately 2.3 nm for electrodeposited Ni-Co/Cu multilayers (the magnetic layer had a Ni:Co ratio of 1:2 and it contained also about 5 at.% Cu). It should be noted that they also applied a much more positive Cu deposition potential (−0.26 V vs. SCE) than the electrochemically optimized value. Therefore, based on the magnetic layer composition, their actual spacer thicknesses can be estimated to be about 0.7 nm larger than the reported nominal values, although we will use their nominal *t*_Cu_ values. Thus, the two GMR peaks shown in their work appear at different spacer thicknesses with respect to the values known from studies on related physically deposited multilayers with relevant Ni:Co ratios [89] and this is a first indication that the two peaks do not correspond to an oscillatory GMR behavior. In addition, their multilayer with *t*_Cu_ = 1 nm exhibits MR(*H*) curves which are dominated by the SPM contribution to the GMR as is evident from the shape and high saturation field of the reported magnetoresistance curve [87,88]. The high remanence of the magnetization [87] also speaks for the absence of an AF coupling. Therefore, their “first peak” is not due to a GMR_FM_ term, so it is not a peak of the oscillatory GMR. The further increase in the GMR for larger *t*_Cu_ values, up to a GMR maximum, corresponds to the generally observed behavior of uncoupled magnetic layers (see, e.g., Ref. [47] and the present work), as evidenced by the hysteresis of both the *MR*(*H*) and *M*(*H*) curves at *t*_Cu_ = 2.3 nm and by the saturation of the magnetoresistance in relatively low magnetic fields.

An apparent oscillatory GMR was also reported for Ni-Co/Cu multilayers electrodeposited on (100) oriented single-crystal Cu substrates [83]. A GMR peak was observed at *t*_Cu_ = 0.7 nm nominal spacer thickness and a much smaller peak at approximately *t*_Cu_ = 2 nm. As the Cu deposition potential was much more positive (–0.2 V vs. SCE) than the electrochemically optimized value, the large first GMR peak was due to a GMR_SPM_ contribution, as evidenced by the shape and non-saturating character of the reported *MR*(*H*) curve, and not due to an oscillatory behavior of the GMR_FM_ term. Due to the too positive Cu deposition potential applied for depositing these multilayers with Co-rich magnetic layers, the actual Cu layer thicknesses are larger, by about 1 nm, than the nominal ones, in light of previous discussions. Therefore, the observed peak positions are definitely not at the spacer thicknesses observed for Ni-Co/Cu multilayers with oscillatory GMR [89].

### 6.3. Increasing GMR with Increasing Spacer Thickness

In the third group of reports on the GMR of electrodeposited Ni-Co/Cu multilayers [52,84,86,90], the GMR was found to be very small, or even zero, for low spacer thicknesses and then gradually increased with the increasing spacer thickness. Qualitatively, these results are in agreement with the present work and also with the results reported for Co/Cu multilayers electrodeposited under controlled conditions [47]. In the following, we will make a quantitative comparison with the present results and discuss the origin of the differences.

**Figure 17:**
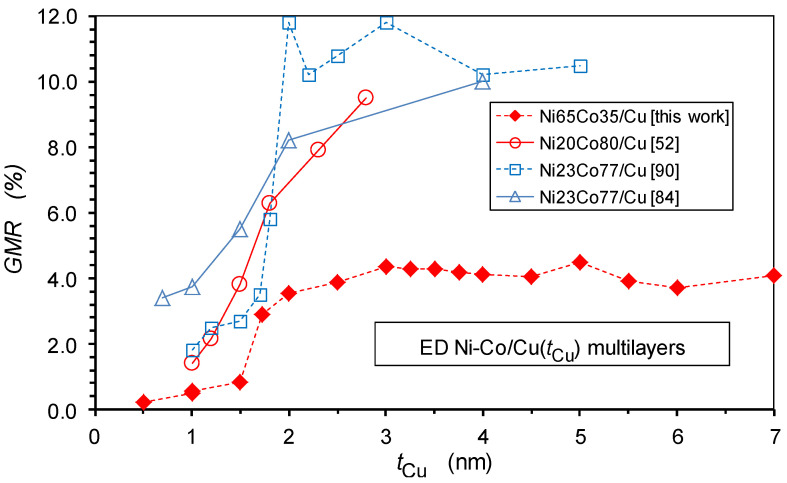
Comparison of the evolution of the *GMR* with spacer layer thickness *t*_Cu_ for the present Ni_65_Co_35_/Cu multilayers and for three other Ni-Co/Cu multilayer studies [52,84,90].

For this purpose, Figure 17 shows our results, together with the results of three previous reports [52,84,90]. The GMR values for large spacer thicknesses in the previous reports are larger than our data by a factor of approximately two. The reason for this is primarily the much larger Co content in the previously studied Ni-Co/Cu multilayers, and this corresponds to the variation of the GMR magnitude with the composition in physically deposited Ni-Co/Cu multilayers exhibiting an oscillatory GMR behavior [89]. The real difference in the behavior of the present result from the previous reports is, however, in the variation of GMR from small spacer thicknesses until GMR saturation is more or less achieved.

It should be noted that only the present data and the data of Ref. [52] were obtained on Ni-Co/Cu multilayers prepared with optimized Cu deposition potential, i.e., the layer thicknesses are the real values only in these cases. For the other two reports [84,90], this was not the case as a potential of –0.3 V vs. SCE was used for Cu deposition and this implies that the true Cu layer thicknesses are larger than the displayed nominal spacer thicknesses for these latter data by about 1 nm.

If we now compare, in Figure 17, our previous results [52] (circles) with the present data (diamonds), it can be seen that the transition from low GMR to high GMR was less sharp in the previous study. This can be due to the rather rough substrate (mechanically polished Ti sheet) used in the previous study [52], whereas in the present work a much smoother substrate was used (Si wafer with evaporated thin Cr and Cu layers with final height fluctuations not larger than 3 nm [57]). It can be expected that on a very smooth substrate, the transition of the Cu layer growth from an island-like behavior to the formation of continuous Cu layers can occur in a relatively sharp manner and at fairly low average Cu thicknesses in comparison with the case of a rough substrate where this transition is smeared out.

In the work of O’Keeffe et al. [84], a relatively smooth substrate was also used (Si electrodeposited with a 10-nm-thick Ni-Co-Cu layer), and the transition from low GMR to high GMR still appears to be rather smeared out (triangles in Figure 17). This can be partially ascribed to the application of a too positive Cu deposition potential as this leads to a partial dissolution of the previously deposited magnetic layer and this is accompanied with an unavoidable surface roughening. With the lack of a reported surface roughness evaluation of the 10-nm-thick Ni-Co-Cu strike layer [84], we cannot be certain that the coverage was continuous on the Si substrate and whether this may have also contributed to the observed smeared transition. At the same time, SPM regions are also formed at low spacer thicknesses, as evidenced by the shape of the *MR*(*H*) curves reported for the lowest nominal spacer thickness [84]. At the lowest spacer thicknesses, the relatively large GMR is actually due to the GMR_SPM_ term.

Finally, the results of Kasyutich et al. [90] are particularly interesting (square symbols in Figure 17). These data were obtained on Ni-Co/Cu multilayers also at a too positive deposition potential, as in Ref. [84], however, electrodeposition was carried out directly onto n-doped GaAs(001) single crystal substrates. The sharp GMR transition suggests that nucleation and growth on this extremely smooth substrate is so favored that it can even eliminate the roughening effect due to the magnetic layer dissolution. An XRD study [90] revealed a particularly good multilayer structure with clear satellite reflections. Nevertheless, the transition is, by considering the true spacer thicknesses, not at about *t*_Cu_ = 2 nm, but at least by 1 nm higher (at about *t*_Cu_ = 3 nm) than the transition for the present Ni-Co/Cu multilayers.

**Figure 18:**
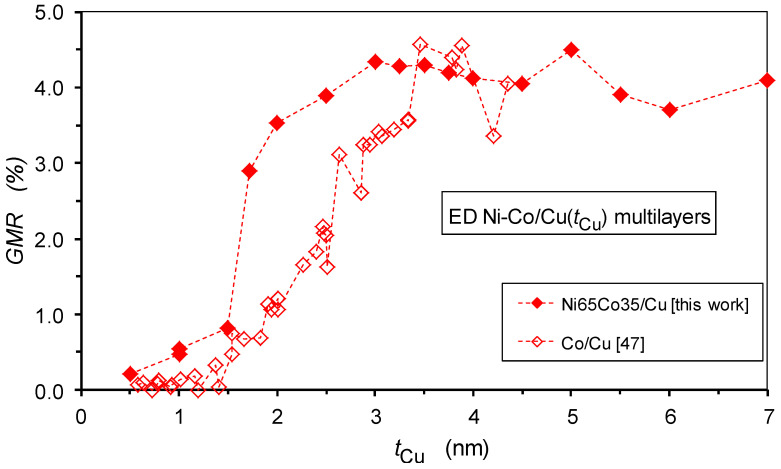
Comparison of the evolution of the *GMR* with spacer layer thickness *t*_Cu_ for the present Ni_65_Co_35_/Cu multilayers and for our previous Co/Cu multilayer study [47].

This “delayed” transition can arise from the different Co contents in the magnetic layers of the two studies (present work and Ref. [90]), as it can be explained with the help of Figure 18, where the present results on Ni_65_Co_35_/Cu multilayers are compared with previous results on Co/Cu multilayers [47]. Both multilayers (Ni_65_Co_35_/Cu and Co/Cu) were prepared in our laboratory under identical conditions (same pulse combination and optimized Cu deposition potential, same bath formulation and same kind of substrate), only the Co content of the magnetic layers was different. The present Ni_65_Co_35_/Cu multilayers show a sharp transition from island-like Cu growth to continuous Cu layer formation with increasing average Cu layer thickness whereas this transition is smeared out in the Co/ Cu multilayers [47].

To understand this difference, we should first consider the layer formation process in these multilayers. At low coverages, the Cu layer formation on the Co and Ni-Co layers proceeds with the nucleation of small islands (so-called Volmer-Weber growth mode [91]). This is partly due to the larger atomic size of Cu with respect to Ni and Co, in addition to the lack of a large attractive (Ni,Co)-Cu atomic interaction compared to the Cu-Cu one. However, the nucleation of Cu islands is somewhat easier on a Ni-Co surface with sufficiently high Ni content compared to a pure Co surface, which can be explained by the complete miscibility of Ni and Cu as opposed to the immiscibility of Co and Cu. Therefore, on a Ni_65_Co_35_ surface, an initial Cu layer can nucleate with islands of larger lateral sizes than on a pure Co surface; thus, the Cu layer can become continuous by the coalescence of Cu islands at a smaller average Cu coverage on a Ni-Co alloy surface. The nucleation of Cu on pure Co is so effectively hindered that the transition to a continuous Cu layer is strongly smeared out in contrast to the more favored nucleation on a Ni_65_Co_35_ surface, where this transition can occur in a fairly narrow Cu layer thickness range as demonstrated by the present results.

It is noted that for electrodeposited Fe5Co95/Cu multilayers [51], the GMR vs. *t*_Cu_ behavior was very similar to that observed for Co/Cu [47], which is shown in Figure 18. This means that a small amount of Fe in the magnetic layer did not have a significant influence on the spacer layer nucleation. Similar results were obtained for the electrodeposited Ni-Co-Cu/Cu multilayers studied by Cavallotti et al. [86], where the magnetic layer had a Ni:Co ratio of 4:96 and it also contained 30 at.% Cu. Furthermore, an even broader transition to the continuous Cu layer regime (the maximum GMR achieved at *t*_Cu_ = 7 nm) was observed [86] than that obtained for our Co/Cu multilayers [47]. The very broad transition in the work of Cavallotti et al. [86] is probably also caused by the fact that both sublayers of their multilayers were deposited under galvanostatic control, in which the so-called exchange reaction [28] between the solution and the deposited magnetic (less noble) atoms during the Cu deposition pulse leads to a completely uncontrolled and random dissolution of magnetic atoms and their replacement by Cu atoms.

At this point, we can now turn back to the comparison (Figure 17) of the present results on Ni_65_Co_35_/Cu multilayers with the data of Ref. [90] on Ni_23_Co_77_/Cu multilayers. In view of the discussion in the previous paragraphs, we can now understand that on a Ni-rich Ni-Co surface, the transition from an island-like Cu layer to a continuous Cu layer should occur at a lower average Cu coverage than that for a Co-rich Ni-Co surface just as observed.

Based on all the considerations put forward above, we can answer an important question; namely, to explain why electrodeposited multilayers do not have a GMR at all (or, at most, have a very small one only) for spacer thicknesses around 1 nm. This question arises because at this Cu layer thickness, the physically deposited multilayers have the largest GMR [3,4,23], particularly those multilayers produced by sputtering, the most powerful technique for preparing thin films and multilayers for spintronic applications

During the sputtering deposition process, very energetic atoms arrive at the substrate. Under certain sputtering conditions, e.g., if the flux of incoming atoms is sufficiently high, when the atoms to be deposited arrive at any place on the substrate surface, they are immediately surrounded by identical atoms in every direction. Thus, at least in limited surface regions with significant lateral sizes, a complete coverage of this surface area will be achieved at relatively low average layer thicknesses, even below the overall monolayer range of the spacer layer. This is well demonstrated by the work of Marrows and Hickey [23], who investigated the GMR of sputtered Co/Cu multilayers in the spacer thickness range between 0.65 nm and 1.1 nm. Thirteen samples were prepared in this range, with increasing Cu layer thickness, in steps of 0.04 nm. Taking into consideration that a 1 nm thickness corresponds to approximately four or five monolayers of Cu, this means that the subsequent samples possessed submonolayer differences in their thickness. The fact that the evolution of GMR in two parallel series for these multilayers (see Figure 1 of Ref. [23]) was very regular with spacer thickness, with a GMR maximum around 0.9 nm, undoubtedly proves that the submonolayer differences between neighboring samples were indeed real. This means that by properly controlling the sputtering process, one can produce a few monolayers-thick continuous Cu layer on the surface of a Co layer.

By contrast, it was shown above that it is not possible to achieve a continuous Cu layer that is a few monolayers thick on the surface of the magnetic layer in the case of electrodeposited Co/Cu and Ni-Co/Cu multilayers. This is due to the different ways in which the incoming ions/atoms become part of the surface during electrodeposition. The incoming ions/atoms have a much lower fluence, they are less energetic and they can undergo diffusion on the surface while searching for an energetically low-lying site through which to enter the crystal. In the case of Cu layer formation, this is particularly critical because the Cu^2+^ ion concentration is very low in the electrolyte solution used for multilayer electrodeposition and the deposition is very slow. Under these conditions, the Cu layer formation starts with the creation of the nuclei of Cu, which need to reach a critical size, after which they will be able to grow [31]. These nuclei are known as islands, the coalescence of which leads to continuous Cu layers. It was mentioned above that the size and chemical interaction differences also hinder the layer-by-layer growth and lead to the Volmer–Weber growth mode [91]. The deposition of the magnetic atoms on the Cu layer surface follows a different route, partly due to the above-mentioned atomic size differences and partly due to the much higher concentration of the ions of the magnetic atoms in the electrolyte and, also, they are deposited at much higher current densities, i.e., at a much higher fluence than Cu. As a consequence of these differences, in opposition to Cu layers, relatively thin (of the order 1 nm or even less) magnetic layers can be produced by electrodeposition in a continuous form.

We can now return to the problem of zero or very small GMR in electrodeposited multilayers with spacer thicknesses of around 1 nm. As discussed above, in these multilayers, the spacer layer in this thickness range is discontinuous (or, punched, even if it reaches the percolation level) due to the island-like growth of the Cu atoms on the surface of the magnetic layers. These discontinuities are subsequently filled up by the magnetic atoms during the subsequent deposition pulse. In this manner, the adjacent magnetic layers become directly bridged via magnetic atoms in the discontinuities of the spacer layer. Depending on the deposition conditions of any multilayer preparation method and on the average spacer thickness, these bridges may be laterally extended in the early stage of the island formation process, as is the case for electrodeposition at low spacer thicknesses or in the later stages of spacer layer growth that may have a diameter of just a few atoms. These are called pinholes and can be observed even in physically deposited multilayers, as demonstrated by Bobo et al. [92] via direct cross-sectional transmission electron microscopy. These authors have also investigated and modeled the influence of the presence of such pinholes on the magnetic properties of magnetic/non-magnetic multilayers, which is also relevant for the magnetoresistance. This is because, via the pinholes, there is a direct FM coupling between adjacent magnetic layers. As a consequence, in regions with a high density of pinholes, the adjacent layer magnetizations are aligned parallel in zero magnetic field; therefore, these regions do not contribute to the large magnetoresistance. According to the modeling of Bobo et al. [92], at a certain density of pinholes, the AF coupling of the pinhole-free structure changes to an orthogonal coupling. If this is the case, the GMR magnitude observed at AF coupling reduces by a factor of approximately two [22,23].

In view of the above discussions, we recall that for electrodeposited multilayers, the spacer layer is certainly discontinuous for spacer thicknesses of approximately 1 nm. The discontinuities that are either narrow pinholes or thicker magnetic bridges are present in such a large concentration that in this spacer thickness range, all of the magnetic layers are ferromagnetically coupled. As a consequence, in zero magnetic field, the magnetizations of the magnetic layers are all aligned parallel and, thus, no GMR contribution can arise upon the application of an external magnetic field. With increasing average spacer layer thickness, the density of such defects creating a direct FM coupling between adjacent magnetic layers gradually diminishes. In this manner, more regions of the multilayers with non-aligned neighboring layer magnetizations appear and a small GMR effect occurs. For completely continuous spacer layers, the magnetic layers become uncoupled and a sizeable GMR effect can be observed. It is believed that this is the real scenario for electrodeposited multilayers prepared under deposition conditions ensuring a proper control of the true layer thicknesses.

Finally, we should make some remarks about the relevance of structural quality on the GMR magnitude of multilayers in terms of the presence or absence of XRD satellite reflections by comparing findings on electrodeposited and sputtered Ni-Co/Cu multilayers as well as the possible influence of texture.

An oscillatory GMR was reported on sputtered Ni-Co/Cu multilayers [55,89,93] for which the XRD patterns indicated an fcc(111) texture, whereas satellite reflections could hardly be observed [89,94], particularly for low spacer thicknesses, for which the GMR was the largest. By contrast, as shown above, the present electrodeposited Ni-Co/Cu multilayers do not exhibit an oscillatory GMR behavior and have a significantly lower GMR than the sputtered counterparts. As demonstrated above, our electrodeposited Ni-Co/Cu multilayers exhibit an fcc(111) texture with clear satellite peaks, even at several higher-order reflections. The electrodeposited Ni-Co/Cu multilayers studied by Kasyutich et al. [90] exhibited larger GMR with fcc(002) texture than our fcc(111)-oriented multilayers, whereas their fcc(111)-textured multilayers had no satellites and possessed almost the same GMR as their fcc(002)–oriented multilayers.

All these features lead to the conclusion that neither an appropriate specific texture, nor the presence of satellites, are a prerequisite for the occurrence of a significant GMR effect in multilayers, irrespective of the preparation methods.

## 7. Conclusions

In the present work, the spacer layer thickness-dependence of GMR in electrodeposited Ni_65_Co_35_/Cu multilayers was studied in order to reveal an oscillatory GMR behavior or the lack of it. The Cu spacer layer was deposited at an electrochemically optimized deposition potential as this condition can ensure that the preset thicknesses of both the magnetic and non-magnetic layers correspond to the true thicknesses, which has not been the case in most previous studies.

All of the multilayers investigated here exhibited a GMR effect, the magnitude of which did not show an oscillatory behavior with spacer layer thickness. A monotonic increase in the GMR was observed with a steep rise of approximately 1.5 nm; then, after 3 nm, it remained nearly constant, with a value of approximately 4%. The high relative remanence of the magnetization hinted at the lack of an AF coupling between the magnetic layers, explaining the absence of an oscillatory GMR. The occurrence of GMR in the lack of an AF coupling can be attributed to the fact that, for spacer thicknesses above 1.5 nm, the adjacent magnetic layers are uncoupled and their magnetization orientations become random. This enables the occurrence of spin-dependent scattering events of conduction electrons traveling via the non-magnetic spacer layers between adjacent magnetic layers with non-aligned magnetizations, which give rise to a GMR effect. The coercive field and magnetoresistance peak field data also corroborate the uncoupled state for high spacer layer thicknesses as both of these parameters progressively approach values that are characteristic of individual magnetic layers. At the same time, the low values of the coercivity parameters at low spacer thicknesses in these multilayers indicated a bulk-like behavior with the absence of a GMR effect, or at most, only a small GMR effect. Both the magnetic and magnetoresistance behavior could be attributed to the discontinuities, which are present in very thin Cu layers (e.g., pinholes) and provide a direct FM coupling between adjacent magnetic layers.

The XRD study on the Ni-Co/Cu multilayers strongly supported the above picture regarding GMR evolution. The XRD patterns revealed a coherent fcc superlattice structure and clear multilayer satellite peaks in all multilayers. From the broadening of the XRD lines, the crystallite size and the microstrain were determined. These data indicated that, at around *t*_Cu_ = 1.5 nm, a sudden increase in crystallite size along the growth direction occurs. This could be related to the fact that, around this spacer layer thickness range, the Cu layer progressively becomes continuous and this enables the formation of coherent superlattice crystallites of at least 100 nm in height, which is much larger than the bilayer thickness.

The present results are qualitatively similar to our previous study on electrodeposited Co/Cu multilayers [47], in which an oscillatory GMR was also not found. However, for Co/Cu multilayers, the transition from zero or small GMR to large GMR occurs over an extended range of spacer thickness. The difference between the behaviors of the two multilayer systems could be explained by the differences in the nucleation and initial layer formation process of Cu on pure Co and on Ni-Co alloys.

Based on the experiences with the present Ni-Co/Cu multilayers and the previously studied Co/Cu multilayers [47], we conducted a critical analysis of previously reported GMR data on electrodeposited Ni-Co/Cu multilayers. Finally, a comparison of the differences in the layer formation between electrodeposited and physically deposited multilayers was also discussed.

## Data Availability

The data that support the findings of this study are available from the authors upon reasonable request.
